# Geopolymer Chemistry and Composition: A Comprehensive Review of Synthesis, Reaction Mechanisms, and Material Properties—Oriented with Sustainable Construction

**DOI:** 10.3390/ma18163823

**Published:** 2025-08-14

**Authors:** Sri Ganesh Kumar Mohan Kumar, John M. Kinuthia, Jonathan Oti, Blessing O. Adeleke

**Affiliations:** Faculty of Computing, Engineering and Science, University of South Wales, Pontypridd CF37 1DL, UK

**Keywords:** geopolymer, alkali-activated materials, thermodynamic modeling, Artificial Neural Networks, aluminosilicate precursors, waste glass, CGFA, alkali-silica reaction, circular economy, sustainable construction, hybrid AAS–ASP systems

## Abstract

Geopolymers are an environmentally sustainable class of low-calcium alkali-activated materials (AAMs), distinct from high-calcium C–A–S–H gel systems. Synthesized from aluminosilicate-rich precursors such as fly ash, metakaolin, slag, waste glass, and coal gasification fly ash (CGFA), geopolymers offer a significantly lower carbon footprint, valorize industrial by-products, and demonstrate superior durability in aggressive environments compared to Ordinary Portland Cement (OPC). Recent advances in thermodynamic modeling and phase chemistry, particularly in CaO–SiO_2_–Al_2_O_3_ systems, are improving precursor selection and mix design optimization, while Artificial Neural Network (ANN) and hybrid ML-thermodynamic approaches show promise for predictive performance assessment. This review critically evaluates geopolymer chemistry and composition, emphasizing precursor reactivity, Si/Al and other molar ratios, activator chemistry, curing regimes, and reaction mechanisms in relation to microstructure and performance. Comparative insights into alkali aluminosilicate (AAS) and aluminosilicate phosphate (ASP) systems, supported by SEM and XRD evidence, are discussed alongside durability challenges, including alkali–silica reaction (ASR) and shrinkage. Emerging applications ranging from advanced pavements and offshore scour protection to slow-release fertilizers and biomedical implants are reviewed within the framework of the United Nations Sustainable Development Goals (SDGs). Identified knowledge gaps include standardization of mix design, LCA-based evaluation of novel precursors, and variability management. Aligning geopolymer technology with circular economy principles, this review consolidates recent progress to guide sustainable construction, waste valorization, and infrastructure resilience.

## 1. Introduction

The construction industry, a cornerstone of global infrastructure [1], is a significant contributor to environmental degradation, responsible for approximately 5–8% of global CO_2_ emissions and extensive resource depletion due to its dependence on Ordinary Portland Cement (OPC) [2,3,4]. This environmental burden has prompted the search for low-carbon alternatives capable of delivering comparable or superior structural performance while reducing ecological impacts [5]. Among these alternatives, geopolymers, low-calcium alkali-activated materials characterized by a three-dimensional N–A–S–H gel network, have gained attention as a sustainable solution [6,7,8]. Geopolymers utilize aluminosilicate-rich by-products such as fly ash, GGBS, and metakaolin, along with emerging precursors like waste glass and coal gasification fly ash (CGFA), aligning with circular economy principles and sustainable infrastructure development [9,10,11,12]. They demonstrate superior fire resistance, chemical stability, and durability in aggressive environments [13,14,15] and directly support several SDGs, including SDG 9 (Industry, Innovation, and Infrastructure), SDG 12 (Responsible Consumption and Production), and SDG 13 (Climate Action) [7,16,17,18,19].

**Figure 1:**
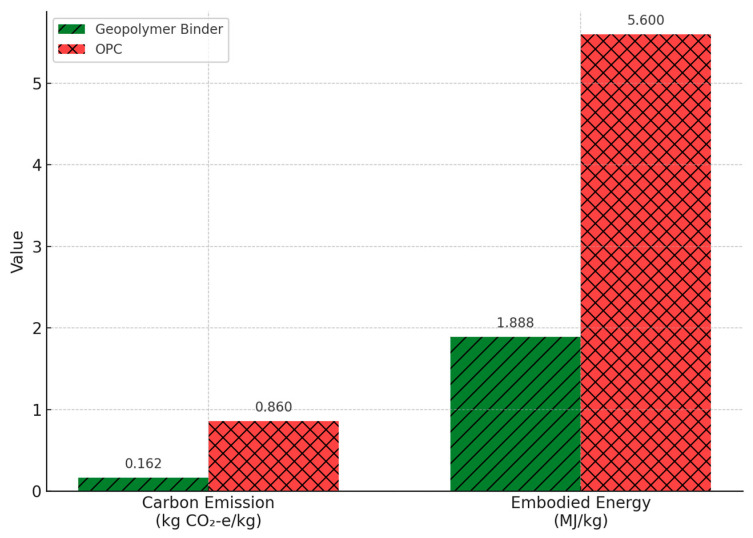
Estimated Environmental impact: Geopolymer vs. OPC [20] [© 2022 by Elsevier Ltd. (Amsterdam, The Netherlands) Licensed under CC BY 4.0].

This review establishes essential molecular-to-macrostructural linkages for advancing sustainable infrastructure materials, with a focused emphasis on underexplored hybrid systems, emerging aluminosilicate precursors, and the need for standardized testing protocols. Unlike previous reviews that primarily concentrate on mechanical or thermal aspects, this work integrates phase chemistry, AI-driven predictive modeling, and cross-sectoral applications including biomedical and environmental engineering thereby repositioning geopolymer technology as a versatile material innovation aligned with the United Nations Sustainable Development Goals (SDGs). Given the fragmented nature of geopolymer research across chemistry, materials science, and civil engineering, this broad-scope review consolidates interdisciplinary insights to support both foundational understanding and future applications across diverse sectors. Table 1 and Figure 1 show that a one-part geopolymer binder has a significantly lower carbon footprint (0.162 kg CO_2_-e/kg) and embodied energy (1.888 MJ/kg) compared to OPC (0.86 kg CO_2_-e/kg; 5.6 MJ/kg), confirming its sustainability potential [20]. These findings align with recent performance and carbon emission studies of geopolymer concrete [21].

### 1.1. Supplementary Cementitious Materials and the Rise of Geopolymers

The growing use of Supplementary Cementitious Materials (SCMs) such as fly ash, GGBS, metakaolin, and rice husk ash has been crucial in reducing clinker demand and associated CO_2_ emissions [3,22,23], with RHA also demonstrating strong pozzolanic reactivity in blended cement systems [24]. However, the decreasing availability of high-quality fly ash due to the global phase-out of coal-fired power plants has accelerated research into alternative precursors [25]. Waste glass, rich in amorphous silica but deficient in alumina, requires supplementary Al sources (e.g., fly ash or calcium sulfoaluminate cement) to ensure adequate geopolymerization [11,26]. Similarly, coal gasification fly ash (CGFA), with its fine particle size and high reactivity, shows promise as a next-generation precursor, although its durability and shrinkage performance remain underexplored [12].

Despite these advances, the global demand for cement remains high, especially in rapidly urbanizing regions, underscoring the need for greener alternatives [16]. Geopolymers can reduce CO_2_ emissions by up to 80% compared to OPC while providing superior fire resistance, lower water absorption, and enhanced durability in aggressive environments [9,13,14,27,28]. These advantages position geopolymers as a strategic material for sustainable infrastructure and low-carbon construction.

### 1.2. Applications of Geopolymers: Terrestrial, Space, and Extraterrestrial

Geopolymers exhibit exceptional versatility, synthesized by activating aluminosilicate-rich precursors such as fly ash and natural minerals with alkaline solutions [10,29,30]. They are applied in diverse sectors, ranging from road construction and heritage building restoration to advanced wastewater treatment systems [31]. Experimental research has extended their potential to extraterrestrial construction, with studies demonstrating compatibility with lunar-like soils, minimal water requirements, and high thermal stability critical attributes for future lunar habitats [32,33,34].

Innovative synergies with thermal regulation technologies have also been reported. For instance, Chen et al. [35] developed a bismuth vanadate/iron oxide yellow composite reflective coating that reduced pavement surface temperatures by >15 °C, indicating potential integration with geopolymer-based pavements for urban heat island mitigation.

Current terrestrial applications span precast elements, pavements, refractory linings, and hazardous waste immobilization [18,31,36]. Their fire and acid resistance make them suitable for offshore structures and advanced wastewater treatment [15,37]. While extraterrestrial applications, including in situ regolith-based lunar habitats, remain experimental [32,33], this review prioritizes terrestrial infrastructure applications relevant to sustainable urbanization and climate-resilient construction.

### 1.3. Material Enhancement and Technology Integration

Recent advances in thermodynamic modeling and phase equilibrium studies, particularly in CaO–SiO_2_–Al_2_O_3_ systems, have improved understanding of reaction product formation and enabled predictive mix design for optimized precursor selection [38,39,40]. In parallel, Artificial Neural Networks (ANNs) and other machine learning models have been increasingly used to predict compressive strength and durability of geopolymers [19,41]. However, hybrid approaches integrating thermodynamic simulations with ML remain underexplored, presenting a key future research opportunity.

Recent advances in geopolymer research have opened many exciting new possibilities. For instance, incorporating nanomaterials has significantly strengthened these materials [42], while integrating natural fibers has enhanced their performance in extreme temperatures [43]. A recent review highlights these advancements, emphasizing sustainable applications and hybrid composite developments in geopolymers [44]. Researchers are also delving into the development of fiber-reinforced geopolymer composites that promise to deliver more resilient infrastructure capable of withstanding the test of time. Geosynthetic reinforcement systems are being digitally modeled to optimize load distribution. Zhao et al. [45] conducted 3D numerical studies comparing different polymer geocells, showing how material type and pocket geometry affect confinement and settlement behavior.

In parallel, cutting-edge technologies such as Artificial Neural Networks (ANNs) are harnessed to design geopolymer mixtures meticulously. These sophisticated AI tools can accurately predict the effects of changes in the mix, such as variations in binder type, curing temperature, or chemical ratios, on critical properties, including strength and workability [41] as depicted in Figure 1. This advance significantly reduces the dependency on time-consuming and resource-intensive laboratory testing.

**Figure 2:**
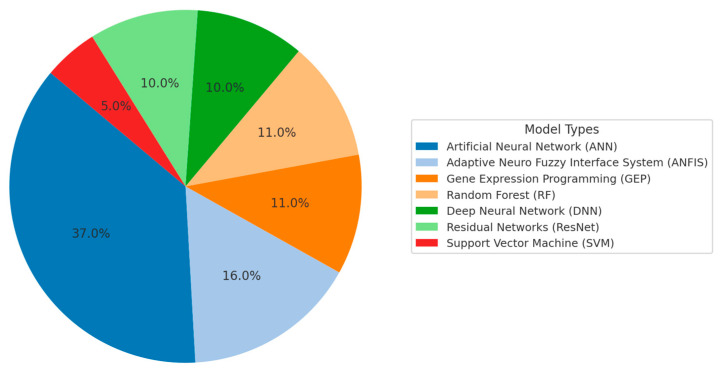
Machine learning techniques used in geopolymer concrete to predict compressive strength [41] [© 2024 by Elsevier Ltd. Licensed under CC BY 4.0].

Figure 2 presents a distribution of machine learning (ML) techniques employed in existing studies for the prediction of the compressive strength of geopolymer concrete. The data indicates that Artificial Neural Networks (ANN) are the most widely used approach, featuring 37% of the reviewed models (7 models). The dominance of ANN can be attributed to its proven ability to model complex nonlinear relationships between input variables and mechanical properties, especially in heterogeneous materials such as geopolymer composites [41].

The second most frequently adopted technique is the Adaptive Neuro-Fuzzy Inference System (ANFIS), accounting for 16% (3 models). ANFIS integrates the learning capabilities of neural networks with the human-like reasoning of fuzzy logic, making it suitable for scenarios with high uncertainty or imprecise input data [41].

Other notable methods include Gene Expression Programming (GEP) and Random Forest (RF), each used in 11% of the studies (2 models each), followed by Residual Networks (ResNet) and Deep Neural Networks (DNN), both deep learning architectures also appearing in 10% of the models (2 models each). These approaches are increasingly adopted for their superior feature extraction capabilities and robustness in handling large datasets [41].

Less frequently applied methods include Support Vector Machine (SVM), which appears in only 5% of the models (1 model). Despite its lower usage, SVM remains a relevant method, particularly when dealing with smaller datasets or high-dimensional input spaces [41].

The distribution indicates a clear research preference for neural network-based models, especially ANN and deep learning variants, due to their ability to effectively capture complex correlations between process variables and the mechanical behavior of geopolymer concrete. However, the diversity of methods reflects an ongoing exploration of hybrid and ensemble ML techniques aimed at improving prediction accuracy and generalization.

**Figure 3:**
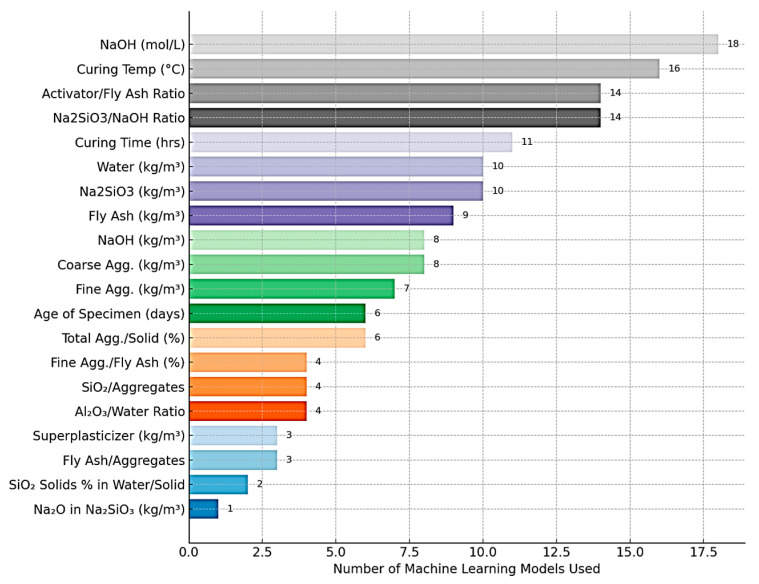
Prevalence of Input Features in Geopolymer Machine Learning Applications [41] [© 2024 by Elsevier Ltd. Licensed under CC BY 4.0].

Figure 3 presents a comprehensive overview of the frequency with which various input parameters have been employed in machine learning (ML) models developed to predict the properties of geopolymer concrete. The analysis is based on the number of models that incorporated each parameter, as represented on the vertical axis, while the horizontal axis enumerates the specific parameters used.

Among the most frequently utilized parameters, sodium hydroxide (NaOH) concentration (mol/L) ranks highest, appearing in 18 ML models. This underscores its fundamental role in governing the dissolution of aluminosilicate precursors and initiating polymerization reactions. Similarly, curing temperature (°C) is included in 16 models, reflecting its critical influence on reaction kinetics and early-age strength development. The alkaline activator-to-fly ash ratio and the Na_2_SiO_3_/NaOH ratio, each cited in 14 models, further emphasize the importance of activator chemistry in determining workability, setting time, and mechanical performance [41].

Parameters such as curing time, water content, and individual activator components Na_2_SiO_3_, fly ash, and NaOH (kg/m^3^) also appear prominently, featuring in 9 to 11 models. These variables are well-known for their impact on the geopolymer matrix’s porosity, compressive strength, and durability [41].

Less frequently used inputs include aggregate properties (e.g., coarse and fine aggregates, total aggregate content), specimen age, and specific chemical constituents such as SiO_2_ and Al_2_O_3_ percentages in fly ash. Additionally, parameters like superplasticizer dosage, fly ash-to-aggregate ratio, and solid content in activators (e.g., Na_2_O in Na_2_SiO_3_) are incorporated into a smaller subset of models [41].

The data clearly indicates that mix design parameters related to the activator system and curing regime are prioritized in predictive modeling, likely due to their dominant influence on the microstructural evolution and performance characteristics of geopolymer concrete. The relatively lower inclusion of aggregate and chemical composition parameters suggests either limited availability of such detailed data in existing studies or a lesser perceived impact on model accuracy within the training datasets used.

### 1.4. Differentiating Geopolymer Systems

Despite extensive research, significant terminological inconsistencies persist in classifying alkali-activated binders. This review explicitly differentiates low-calcium geopolymers from high-calcium alkali-activated materials (AAMs), as summarized in Table 2.

Variations in precursor chemistry ranging from silicate- to phosphate-based systems significantly affect mechanical performance and durability [6,52]. Microstructural studies have shown that gel structure, Ca/Si ratios, and unreacted phases strongly influence strength development and shrinkage behavior [39,53]. However, the lack of standardized terminology and wide variability in industrial by-products sourced from different regions continue to hinder cross-comparability of research findings [54].

### 1.5. Material Variability and Testing Standards

The high variability in industrial by-products and the lack of standardized testing protocols remain critical barriers to large-scale commercialization of geopolymers [25,54,55]. Durability concerns particularly alkali–silica reaction (ASR) and drying shrinkage require urgent attention, as high-alkali activators and reactive aggregates significantly increase ASR susceptibility [14,15,27]. Recent studies highlight mitigation strategies, including optimized activator concentrations, incorporation of supplementary Al-rich precursors, and use of blended aggregate systems to reduce ASR and shrinkage [15,27,53,56]. This review synthesizes emerging insights on optimal precursor selection, activator chemistry, curing regimes, and mix design parameters that directly influence microstructural stability and durability [57]. Aligning research with global sustainability initiatives and adopting Life Cycle Assessment (LCA)-based frameworks can accelerate standardization and regulatory acceptance, promoting geopolymers as mainstream low-carbon construction materials [58].

### 1.6. Alignment with Key SDG’s

Geopolymers significantly contribute to circular economy principles and align with multiple United Nations Sustainable Development Goals (SDGs). They enable substantial CO_2_ reduction, valorize industrial by-products, and provide safe immobilization of hazardous wastes, while enhancing climate-resilient infrastructure systems [18,59].

Table 3 outlines the SDGs most impacted by geopolymer technology, highlighting key contributions and critical research gaps that need to be addressed for broader adoption.

Rooted in green chemistry principles, geopolymer technology supports the UN’s 2030 Sustainable Development Agenda by transforming industrial wastes into durable, low-carbon construction materials [57]. Several SDGs recognize green chemistry as a critical driver for tackling environmental, social, and economic challenges, positioning geopolymers as a key material innovation [17].

### 1.7. Sector Specific Contributions

Geopolymers extend their impact beyond conventional construction, contributing to multiple SDGs through sector-specific innovations. In the built environment, geopolymers foster sustainable urban development (SDG 11), drive industrial innovation, and enhance infrastructure resilience (SDG 9), while achieving substantial CO_2_ emission reductions (SDG 13) [15,57,61]. In the energy sector, fly ash– and slag–based geopolymer systems are increasingly applied in geo-energy well stabilization and gas treatment, directly supporting affordable and clean energy targets (SDG 7) [62]. Emerging studies highlight their potential in advanced catalysis and electronic component manufacturing, further strengthening their role in industrial innovation (SDG 9) [63]. In agriculture, geopolymer-based slow-release fertilizers and soil conditioners improve crop productivity and support sustainable land management, directly aligning with food security (SDG 2) and ecosystem restoration goals (SDG 15) [64,65].

### 1.8. Biomedical and Environmental Applications

Geopolymers are increasingly explored in biomedical and environmental engineering, demonstrating significant multifunctional potential. In healthcare, bioactive geopolymer composites are being investigated for bone tissue regeneration and controlled drug delivery systems, offering potential breakthroughs in improving health outcomes (SDG 3) [63]. In marine engineering, applications range from artificial reef construction to offshore scour mitigation. Fly ash–slag alkali-activated grouts have been shown to improve soil cohesion and substantially reduce scour depth around monopile foundations [66,67]. In water and waste management, their high chemical stability enables safe encapsulation of radioactive waste and development of advanced water purification technologies, directly contributing to clean water and sanitation goals (SDG 6) [18].

### 1.9. The Way Forward: Adoption and Integration

Geopolymers provide a versatile cross-sectoral pathway to sustainable development, spanning construction, energy, agriculture, and biomedical applications [7,19]. However, large-scale adoption remains constrained by the lack of standardized testing protocols, limited policy incentives, and the need for stronger interdisciplinary collaboration to address precursor variability and performance inconsistency [25,55,68]. Aligning geopolymer research with global sustainability frameworks and integrating Life Cycle Assessment (LCA)-based policies are critical for establishing geopolymers as mainstream low-carbon construction materials, strengthening their role in climate resilience and innovation-driven growth [17,58,69]. Future collaborative frameworks among industry, academia, and policymakers are critical to scale up pilot projects and establish geopolymers as mainstream sustainable binders by 2030.

## 2. Understanding Geopolymers: Origins and Chemistry

### 2.1. Historical Origins and Definitions

The concept of geopolymers was pioneered by Joseph Davidovits in the 1970s [6,70], representing a paradigm shift in low-carbon construction materials. Defined as inorganic aluminosilicate polymers formed by the alkali or acidic activation of aluminosilicate-rich precursors at temperatures typically below 100 °C [29,71], geopolymers offer an eco-friendly alternative to Portland cement. Their low-temperature synthesis and near-zero CO_2_ emissions provide a critical pathway toward reducing the carbon footprint of construction materials [2,9,72]. Furthermore, their ability to incorporate industrial and agricultural waste aligns with circular economy principles, as highlighted in recent sustainability reviews [16,57,73].

### 2.2. Molecular Structure and Polymerization Process

Geopolymers are amorphous to semi-crystalline aluminosilicate frameworks, wherein Si–O–Al bonds form robust three-dimensional networks [8,46]. This network arises from geopolymerization, a polycondensation reaction occurring under alkaline or acidic conditions [6,74]. In alkaline systems, aluminate and silicate species dissolve from precursors and recombine as sialate (–Si–O–Al–O–) and polysialate (–Si–O–Al–O–Si–O–) linkages, stabilized by alkali cations (Na^+^, K^+^) [8,29]. This mechanism yields highly durable, chemically resistant binders with mechanical strengths surpassing many traditional cementitious systems [30,46]. Acidic activation less explored but gaining interest forms aluminosilicate-phosphate networks (–Si–O–Al–O–P–) that enhance thermal stability and dielectric properties [74,75,76]. Recent nanoscale analyses suggest that alkali concentration and Si/Al ratio strongly influence the cross-link density and hence durability [77,78,79].

### 2.3. Types and Structural Variants of Geopolymers

Geopolymers can be broadly classified based on their hardening agents, precursor sources, and functional applications, reflecting the diversity of structural configurations and material performance.

#### 2.3.1. Classification by Hardening Agents and Network Structure

Alkali-Aluminosilicate (AAS) Geopolymers—formed through alkali activation of aluminosilicate precursors, predominantly consisting of repeated –Si–O–Al–O– chains [46,52]. These are the most widely studied for construction applications due to their high compressive strength and durability [31,57,68].

Aluminosilicate Phosphate (ASP) Geopolymers—produced in acidic media (commonly using phosphoric acid) and characterized by structural units such as –Si–O–Al–O–P–, –Si–O–P–O–Al–, or –Al–O–P– [74,75,76]. ASP geopolymers exhibit high thermal stability and chemical resistance, making them suitable for applications in catalysis, refractory linings, and electronic materials [80,81].

Ferro-Sialate Geopolymers—emerging systems where iron substitutes for aluminum within the structural network, forming ferro-sialate linkages such as –Fe–O–Si–O–Al–O– under alkaline conditions and –Si–O–P–O–Si–O–Fe– in acidic environments [82,83]. These are particularly attractive for waste valorization of iron-rich by-products like red mud and lateritic soils [49].

#### 2.3.2. Classification by Precursor Type

The selection of precursors significantly affects reactivity, gel formation, and sustainability outcomes. Precursor-based classification highlights natural aluminosilicates, industrial by-products, and emerging alternative materials:

Natural Precursors—Metakaolin, kaolinite, zeolites, and volcanic ash (pumice, tuff) are primary aluminosilicates rich in reactive SiO_2_ and Al_2_O_3_, contributing to high mechanical performance and refined pore structure [57,84,85,86]. Metakaolin, for example, exhibits rapid dissolution and early strength development [86,87].

Industrial By-products—Fly ash, ground granulated blast furnace slag (GGBFS), red mud, and steel slag are the most utilized due to their waste valorization potential and circular economy benefits [57,72,88]. Fly ash and slag systems predominantly form N–A–S–H and C–A–S–H gels, enhancing durability and reducing carbon emissions by up to 64% compared to Portland cement [72,73,89].

Emerging Precursors—With the decline in high-quality fly ash availability, alternative materials such as coal gasification fly ash (CGFA), waste glass, bamboo leaf ash (BLA), rice husk ash (RHA), rice straw ash (RSA), and basalt powder are gaining attention [11,12,25,90,91]. Waste glass, with high amorphous silica and intrinsic alkalis, serves both as a reactive precursor and a self-activating agent [11,26]. Agricultural ashes like RHA and BLA are particularly valuable for low-cost, region-specific applications [57,92,93]. Similarly, recycled plastic aggregates are being explored to enhance sustainability and divert polymeric waste from landfills [94].

A rational combination of high-reactivity precursors (e.g., metakaolin) with supplementary waste-derived sources (e.g., slag, red mud) is increasingly recommended to optimize Si/Al and Ca/Si ratios, balancing mechanical performance and sustainability [39,46,57].

#### 2.3.3. Functional Classification by Application

Recent advancements have diversified geopolymer technology into application-specific categories:

Structural and Infrastructure Applications—High-performance fly ash–slag blends are used for precast elements, pavements, and marine infrastructure due to their enhanced durability and low permeability [31,55,68]. Self-compacting and ultra-high performance geopolymer concretes have been developed for specialized structural applications [95,96].

Thermal and Fire-Resistant Applications—Potassium-rich and ASP geopolymers exhibit high refractoriness (>1000 °C) and thermal stability, making them ideal for fire-resistant panels, furnace linings, and thermal insulation [13,74,76].

Environmental and Waste Management Applications—Geopolymers are increasingly applied in immobilizing hazardous wastes (e.g., heavy metals, radioactive waste) due to their dense microstructure and ion-exchange capacity [18,59,97].

Biomedical and Agricultural Applications—Recent studies have explored biocompatible geopolymer composites for bone tissue engineering [98] and agricultural slow-release fertilizers synthesized from agricultural and loess-based precursors [64,65].

Advanced and Emerging Applications—Developments in 3D-printable geopolymers [99,100], nanomodified composites [42,101], and lunar construction materials [32,33] reflect the adaptability of geopolymer chemistry to future construction paradigms.

#### 2.3.4. Hybrid and Composite Variants

Hybrid binders that combine geopolymer chemistry with supplementary fibers or nanoparticles are gaining traction. Fiber-reinforced geopolymers enhance tensile strength and crack resistance [43,53], while nano-SiO_2_ and graphene additions improve densification and microstructural refinement [42,101]. These variants expand the functional scope of geopolymers beyond conventional cement replacement.

### 2.4. How Geopolymerization Works and Its Thermal Properties: Phase Chemistry and Gel Structures

Geopolymers differ fundamentally from traditional Portland cement in their reaction mechanisms and thermal behavior. While Portland cement hardens through hydration, geopolymers undergo polycondensation, forming continuous aluminosilicate frameworks with superior thermal and chemical resistance [8,30].

Stages of Geopolymerization:

1. Dissolution/Depolymerization—aluminosilicate precursors (e.g., fly ash, metakaolin) dissolve in an alkaline or acidic medium, releasing reactive silicate and aluminate species [29,46].
2. Polycondensation—reactive species reorganize into oligomers and polymerize into three-dimensional networks, forming N–A–S–H or K–A–S–H gels in alkaline systems, or aluminosilicate phosphate gels in acidic systems [74,75,76].
3. Gel Hardening and Network Growth—progressive cross-linking yields amorphous or semi-crystalline structures with high mechanical strength and durability [30,47]. Nano-silica addition modifies the hydration behavior of reactive aluminosilicates, improving gel densification and early-age strength [102].

The chemistry depends on precursor type and activating medium: calcium-rich precursors (e.g., GGBS) form hybrid N–A–S–H + C–A–S–H gel systems, accelerating early strength [103,104], while iron-rich sources (e.g., red mud, basalt) can replace Al to form ferro-sialate gels, improving density and thermal stability [82,83].

Thermal Properties and Phase Transformations:

1. Amorphous-to-Crystalline Transition: Sodium-based geopolymers crystallize into nepheline/sodalite at ~500 °C, while potassium-based systems form leucite/kalsilite phases above 1000 °C, making them suitable for refractory applications [7,13,75].
2. Acidic Systems: ASP-based geopolymers (e.g., phosphoric-acid-activated systems) show high thermal stability and low shrinkage, ideal for electrical insulation, catalytic processes, and aerospace uses [74,81].
3. Hybrid/Waste-Derived Systems: Waste glass (WG) and rice husk ash (RHA) refine microstructure and enhance thermal resistance; WG-based systems maintain >90% compressive strength at 250 °C [11,26].

In summary, the polycondensation pathways and thermal phase chemistry determine the structural performance of geopolymers, enabling applications such as fire-resistant panels, thermal insulators, and high-temperature industrial components.

### 2.5. A Sustainable Future with Geopolymers

Geopolymers are increasingly recognized as sustainable construction materials due to their low-temperature synthesis, non-hydration-based hardening, and ability to utilize large volumes of industrial and agricultural waste [7,9,57]. Their production aligns with circular economy principles by valorizing by-products such as fly ash, red mud, and slag, while substantially reducing landfill disposal [16,68].

Life cycle assessments show geopolymers can reduce CO_2_ emissions by 40–80% compared to Portland cement, depending on the precursor and activator type [72,73]. This reduction is attributed to the absence of high-temperature clinkerisation and the recycling of waste streams, contributing to low-carbon infrastructure development [58,73].

Geopolymers also exhibit excellent durability and long-term stability, with resistance to sulfate attack, acid corrosion, and high-temperature degradation, making them suitable for marine infrastructure, road maintenance, and nuclear waste immobilization [37,68,88]. Additionally, emerging studies highlight their role in renewable energy systems, fire-resistant components, and 3D-printed low-carbon buildings [31,42,99].

In conclusion, geopolymers represent a transformative alternative to conventional cementitious systems, directly supporting global climate goals and UNEP Green Chemistry Frameworks [17]. With continued research into low-cost activators (e.g., RHA-derived silicates [104]) and performance optimization, geopolymers are expected to play a central role in achieving net-zero construction targets [57,73].

### 2.6. Aluminosilicate Materials: Classification and Functional Roles in Geopolymer Technology

Aluminosilicate materials are the primary precursors in geopolymer technology, derived from natural sources (calcined clays, zeolites, volcanic ash, pumice) and industrial by-products (fly ash, red mud, slag, mine tailings) [25,57]. Industrial sources are prioritized in sustainable construction due to their waste valorization potential, consistent with circular economy principles [16,68]. Selection criteria include amorphous phase content, Si/Al ratio, and environmental sustainability, which directly influence N–A–S–H and C–A–S–H gel formation, durability, and early strength development [78,84]. Standardizing these criteria reduces variability and improves reproducibility in mix designs [105].

**Figure 4:**
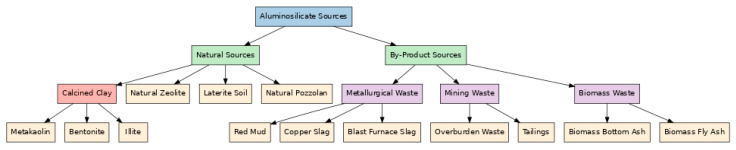
A schematic representation of the Aluminosilicate Sources [43] [with permission from Elsevier].

Figure 4 illustrates the diverse sources of aluminosilicate materials commonly used in geopolymer synthesis. These are broadly classified into natural sources such as calcined clay and natural zeolite and by-product sources derived from various industrial sectors including metallurgy, mining, and biomass processing.

Aluminosilicate sources are broadly categorized into: (i) true precursors highly reactive and amorphous (e.g., metakaolin, fly ash, GGBS); and (ii) auxiliary components low-reactive or crystalline fillers (e.g., quartz, dolomite, silica fume), as elaborated in Section 2.7 and Section 2.8 [57,84]. This distinction is crucial for optimizing precursor reactivity, controlling gel chemistry (via thermodynamic modeling), and predicting long-term performance [57,84].

Natural sources primarily include calcined clay (metakaolin), natural zeolite, volcanic ash, pumice, and other clay minerals such as kaolinite, laterite, and montmorillonite [84]. These materials are rich in reactive amorphous alumina (Al_2_O_3_) and silica (SiO_2_), which are essential for forming geopolymeric gels that impart mechanical strength, chemical stability, and long-term durability to the resulting composites [30,106].

Industrial by-product sources encompass fly ash, ground granulated blast furnace slag (GGBFS), red mud, fayalite slag, mine tailings (e.g., copper, hematite), and basalt powder [57,68]. These materials not only contribute to the formation of gel phases such as N–A–S–H and C–A–S–H but also align with circular economy principles by valorizing high-volume industrial waste streams, thereby reducing CO_2_ emissions by up to 64% compared to traditional Portland cement [49,58,72,88].

With the global decline in high-quality fly ash due to the phase-out of coal-fired power plants, emerging precursors such as coal gasification fly ash (CGFA) and waste glass are gaining attention [11,12,25]. CGFA, with its fine particle size and high reactivity, offers significant potential for next-generation geopolymers, while waste glass, rich in amorphous silica, has shown promise not only as a reactive precursor but also as a high-temperature stabilizer [11,26]. However, their low alumina (Al_2_O_3_) content necessitates blending with Al-rich sources such as fly ash or calcium sulfoaluminate cement to ensure effective geopolymerization [11,26].

The rational selection and combination of these materials considering their chemical composition (Si/Al and Ca content), particle morphology, and amorphous phase proportion is essential for optimizing precursor reactivity and long-term durability [38,57]. This approach reinforces sustainability by reducing reliance on conventional cementitious materials and supporting waste valorization strategies, which are key to achieving low-carbon construction [57,73].

### 2.7. Material Constituents of Geopolymeric Systems

#### 2.7.1. Aluminosilicate

The material constituents of geopolymeric systems are primarily determined by the aluminosilicate precursors and supplementary components that govern reactivity, gel formation, and long-term durability [46,57]. Industrial by-products such as fly ash (FA), ground granulated blast furnace slag (GGBS), and red mud are most used, either individually or in blended systems with natural aluminosilicate sources like metakaolin, to enhance mechanical and chemical performance [57,68,73].

#### 2.7.2. Fly Ash (FA)

Fly ash is a by-product of coal-fired power plants, formed during the high-temperature combustion of pulverized coal. Mineral matter undergoes thermal decomposition, producing fine spherical particles entrained in flue gases, while heavier bottom ash settles within the combustion chamber. Collected through electrostatic precipitators or bag filters, fly ash is rich in reactive amorphous silica (SiO_2_) and alumina (Al_2_O_3_), making it an ideal aluminosilicate precursor for geopolymerization [2,107].

In traditional cementitious systems, fly ash undergoes pozzolanic reactions with calcium hydroxide to form cementitious compounds. In geopolymer systems, however, it reacts with alkaline activators (e.g., NaOH, Na_2_SiO_3_) to form N–A–S–H gels, contributing significantly to strength and chemical stability [107,108]. The spherical morphology of fly ash particles enhances workability and flowability, while its high amorphous content accelerates dissolution and polymerization [108]. However, global fly ash production (>1 billion tons annually) poses serious challenges, further justifying its valorization in sustainable construction [2,25].

**Figure 5:**
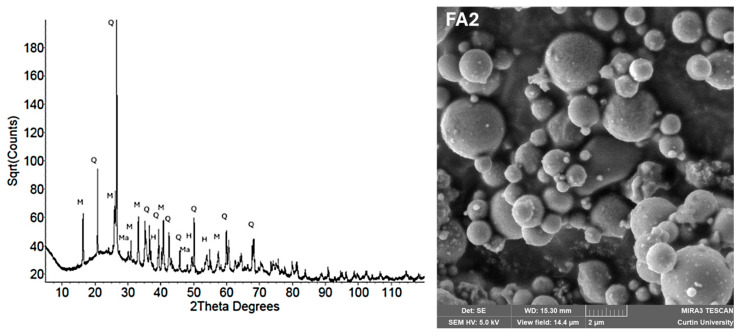
Fly Ash (FA)—XRD Analysis and SEM Analysis [108] (© 2025 by the authors. Licensed under CC BY 4.0).

Figure 5 presents a scanning electron microscope (SEM) image and an X-ray diffraction (XRD) pattern of fly ash. The SEM image confirms its smooth spherical morphology, and the XRD profile indicates dominant amorphous phases critical for geopolymerization, alongside minor crystalline impurities [108].

#### 2.7.3. Ground Granulated Blast Furnace Slag (GGBS)

GGBS is a high-calcium industrial by-product from iron and steel manufacturing, formed by quenching molten slag at ~1500 °C, producing vitrified granules that are then ground into fine powder [96,104]. Its glassy structure and high CaO content enables latent hydraulic activity, making it a key supplementary cementitious material (SCM) for blended binders [96].

In geopolymer systems, GGBS contributes significantly to early strength and durability by forming C–A–S–H gels that complement N–A–S–H networks [104,109]. Dai et al. [103] reported that GGBS-based systems achieved ~30% higher early compressive strength compared to fly ash geopolymers, consistent with their high CaO content and reactive amorphous phase. As an abundant steel industry by-product, GGBS valorization aligns with circular economy principles and addresses industrial waste disposal challenges [25].

**Figure 6:**
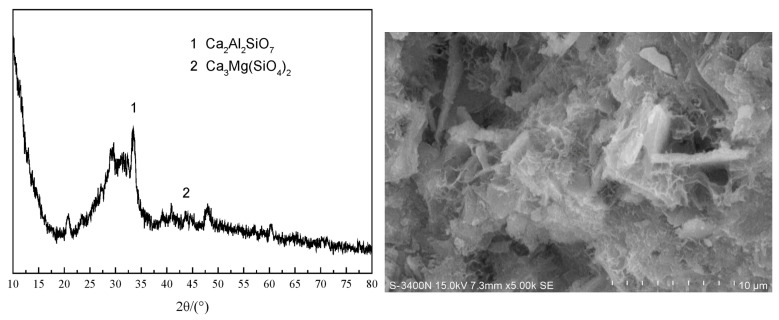
Ground Granulated Blast Furnace Slag (GGBS)—XRD Analysis and SEM Analysis [103] (© 2025 by the authors. Licensed under CC BY 4.0).

Figure 6 presents the scanning electron microscope (SEM) image and X-ray diffraction (XRD) pattern of GGBS. The SEM analysis reveals the irregular morphology of GGBS particles and confirms their largely amorphous nature, which correlates with high chemical reactivity often exceeding that of fly ash (FA). The XRD pattern further substantiates this enhanced reactivity through the identification of characteristic broad humps indicative of the amorphous phase. However, while GGBS improves the binder’s cementitious performance, the irregular particle shape may adversely affect the flowability of fresh mixtures.

#### 2.7.4. Metakaolin (MK)

Metakaolin (MK) is a thermally activated clay produced by calcining kaolinite (Al_2_Si_2_O_5_(OH)_4_) at 600–800 °C, transforming it into a reactive amorphous aluminosilicate phase [86]. Its layered alumina–silica structure (Al(O,OH)_6_ linked with SiO_4_ tetrahedra) imparts high pozzolanic reactivity.

In geopolymer systems, MK is valued for its high purity and consistent reactivity, contributing to rapid early strength development and a refined pore structure [86,87]. Studies have shown that MK-blended geopolymers achieve significantly higher compressive strength compared to fly ash-only systems due to its high reactive alumina content [108].

Kaolin is widely available globally, with major producers including the United States, Germany, China, Brazil, South Korea, and Iran, making MK a reliable and accessible precursor for high-performance geopolymers [87]. Although not derived from industrial waste streams like FA or GGBS, its controlled chemical composition makes it particularly suitable where waste-derived precursors show variability or limited availability.

**Figure 7:**
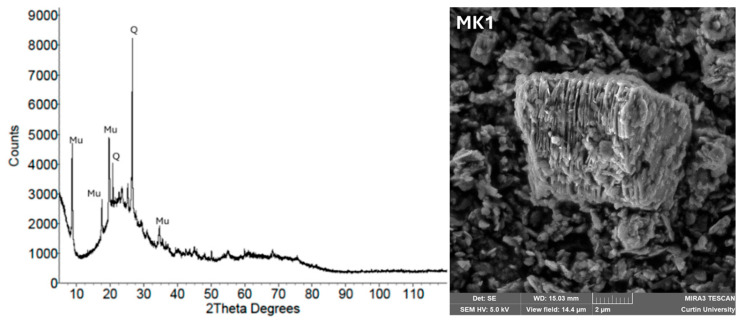
Metakaolin (MK)—XRD Analysis and SEM Analysis [108] (© 2025 by the authors. Licensed under CC BY 4.0).

Figure 7 presents a scanning electron microscope (SEM) image and an X-ray diffraction (XRD) pattern of metakaolin. The SEM image reveals a plate-like morphology, which promotes effective dispersion and reactivity during geopolymerization. The XRD pattern displays characteristic peaks of quartz and muscovite within the 2θ range of 10° to 70°, indicating a semi-crystalline structure consistent with thermally activated kaolinite.

#### 2.7.5. Red Mud (RM)

Red Mud, a by-product of alumina extraction (Bayer process), is rich in Al_2_O_3_ (~30%) and Fe_2_O_3_ (~8–9%) [51], making it suitable for alkali activation. Its reactive alumina contributes to N–A–S–H gel formation, while Fe_2_O_3_ promotes (Fe)–A–S–H gels, improving density and thermal stability [49].

Incorporating Red Mud in geopolymers mitigates hazardous waste disposal issues and aligns with circular economy goals by replacing virgin raw materials and reducing CO_2_ emissions by up to 64% compared to OPC [72,88].

Comparative SEM/XRD studies have shown angular particles and crystalline phases (hematite, gibbsite) embedded within an amorphous matrix [51], influencing reactivity and requiring optimized mix designs (e.g., blending with fly ash or slag) to control workability and shrinkage.

#### 2.7.6. Waste Glass (WG)

Waste Glass (WG), rich in reactive SiO_2_ (~70%) and intrinsic alkalis (Na_2_O~9.7%), serves as both a precursor and a self-activating agent [11]. However, due to its low intrinsic Al_2_O_3_ content [110], WG-based geopolymers typically require co-precursors such as fly ash, metakaolin, or calcium sulfoaluminate cement to ensure adequate Al availability for tetrahedral framework formation and improved structural integrity [26]. Dai et al. [26] further reported that WG powder enhances high-temperature durability, maintaining >90% compressive strength after curing at 250 °C, making it a suitable additive for thermally stable geopolymer systems.

Although fine WGP can help mitigate alkali–silica reaction (ASR) expansion, its high alkali content (particularly Na_2_O) requires careful proportioning to avoid deleterious expansion or shrinkage. Studies in cement pastes confirm that while WGP improves high-temperature stability, its use in geopolymer systems demands systematic durability evaluation, and supplementary alumina sources are recommended to maintain long-term mechanical and chemical stability [26].

SEM and XRD evidence (from recent studies) indicate WG particles are angular and amorphous, with characteristic broad SiO_2_ humps and minor crystalline quartz peaks [11], supporting its high reactivity in alkaline environments.

#### 2.7.7. Coal Gasification Fly Ash (CGFA)

Coal Gasification Fly Ash (CGFA), generated from integrated gasification combined cycle (IGCC) processes, is a promising alternative to conventional coal combustion fly ash, especially given the declining availability of power plant-derived fly ash and slag. CGFA typically contains high reactive SiO_2_ (~60–62%) and moderate Al_2_O_3_ (~24–25%), comparable to Class F fly ash, but with lower CaO (~1–2%) [12]. This composition favors the formation of N–A–S–H gels in low-calcium alkali aluminosilicate (AAS) systems. However, its trace heavy metal content (e.g., Pb, As) requires encapsulation strategies to ensure environmental safety.

Recent SEM and XRD analyses reveal that CGFA particles are mostly spherical to sub-angular, with a mixed amorphous–crystalline structure. Fine grinding and thermal pretreatment have been shown to enhance its reactivity and dissolution kinetics, making it suitable for hybrid systems when blended with high-CaO precursors (e.g., GGBS or phosphorus slag) to improve workability and early-age strength [12].

Its utilization not only aligns with circular economy goals by valorizing industrial by-products but also offers a transitional pathway for maintaining aluminosilicate supply as coal power plants are phased out.

#### 2.7.8. Agricultural Ashes (Rice Husk Ash, Bagasse Ash, Bamboo Leaf Ash, Etc.)

Agricultural ashes (Rice Husk Ash—RHA, Sugarcane Bagasse Ash—SBA, Bamboo Leaf Ash—BLA) are emerging sustainable precursors, with high amorphous silica content (>70%) and moderate alkali levels [90,93]. Controlled calcination improves pozzolanic reactivity, making them ideal for low-cost, region-specific geopolymer binders [68,92,111].

RHA-derived sodium silicate solutions have been synthesized through eco-friendly processes [104,109], offering a green alternative to commercial activators. SEM images of RHA reveal porous, honeycomb-like morphologies, enhancing dissolution kinetics [108].

#### 2.7.9. Basalt Powder and Other Emerging Precursors

Basalt Powder (BP), rich in SiO_2_ (~45%) and Fe_2_O_3_ (~15%), and Rice Straw Ash (RSA) (>70% SiO_2_) are gaining attention as regionally available, low-cost binders [91,112]. BP’s iron content can influence microstructural densification, while RSA contributes to lightweight, thermally resistant geopolymer composites. Their use promotes localized, low-carbon construction practices.

Emerging studies show that BP has angular crystalline particles, while RSA shows amorphous silica-rich phases; both require fine grinding to enhance reactivity [91,112].

Table 4 presents a comparative microstructural evaluation of major industrial and emerging aluminosilicate precursors based on scanning electron microscopy (SEM) and X-ray diffraction (XRD) analyses. The SEM observations capture distinct morphological features such as particle shape, surface texture, and porosity that influence precursor reactivity, water demand, and workability. Complementary XRD data provide insights into the amorphous and crystalline phase assemblages, highlighting the reactive potential and gel formation tendencies of each material. Together, these observations link precursor characteristics to their microstructural implications in geopolymer systems, offering a basis for tailoring mix designs to balance workability, setting kinetics, and long-term durability.

### 2.8. True Precursors vs. Auxiliary Components

True precursors are characterized by high contents of reactive and amorphous alumina (Al_2_O_3_) and silica (SiO_2_), which are critical for initiating geopolymerization and forming robust geopolymeric gels [46,106]. These materials govern the binder’s mechanical strength, durability, and long-term stability [30]. Natural primary precursors include kaolinite, metakaolin, pumice, zeolite, mullite, laterite, and montmorillonite, while secondary precursors derived from industrial by-products such as fly ash, ground granulated blast furnace slag (GGBS), steel slag, copper and hematite tailings, red mud, palm oil fuel ash, and basalt support circular economy principles by transforming waste into high-value reactive binders [57]. Emerging secondary precursors such as coal gasification fly ash (CGFA), rice husk ash (RHA), bamboo leaf ash (BLA), basalt powder, and waste glass are increasingly recognized for their reactive silica content and are gaining attention for sustainable geopolymer production [11,12,26,90,91].

In contrast, auxiliary components frequently misclassified as primary precursors contain low or insufficient amorphous alumina and silica [46]. They include mineral-based materials such as silica fume, dolomite, feldspar, vermiculite, and quartz, as well as agricultural by-products like corn cob ash and coconut husk ash [57,68,92]. Although limited in direct reactivity, these materials play significant roles as performance enhancers for example, acting as fillers, nucleation sites, silicate-adjusting agents, or improving rheology and dimensional stability. The synthesis of sodium silicate activators from waste glass and RHA further demonstrates the auxiliary role of these materials in reducing reliance on commercial activators [104,109].

Auxiliary materials indirectly enhance tensile strength, toughness, and chemical resistance, allowing for fine-tuning of mix performance in specialized applications [68]. However, their contribution remains secondary compared to the fundamental role of true precursors in gel formation.

Correct classification of true precursors versus auxiliary components is essential for standardized mix design and reproducible performance, directly addressing one of the major barriers to commercialization highlighted by recent reviews [57,84,105].

Moreover, the effectiveness of geopolymer synthesis is strongly influenced by the chemical makeup of the selected raw materials. Commercially available metakaolin and volcanic ash, known for their high concentrations of reactive silica (SiO_2_) and alumina (Al_2_O_3_), are commonly used to improve structural strength and long-term durability [57]. Materials with significant calcium oxide (CaO) content, such as water-quenched slag, contribute to the development of calcium silicate hydrate (C–S–H) phases and polymeric gels, further boosting performance [38]. Additionally, iron oxides (Fe_2_O_3_), present in materials like red mud and laterite, can influence the coloration and microstructure of the final product [49].

The integration of fly ash, slag, and red mud as secondary materials serves a dual function waste valorization and enhancement of geopolymer properties: promoting circular economy principles by minimizing waste and substantially reducing carbon emissions (by 64%) compared to traditional Portland cement [72,88]. Locally sourced materials not only reduce transportation emissions but also improve cost efficiency and adaptability, reinforcing geopolymer technology as a cornerstone of sustainable construction practices [73].

Waste glass, in particular, has demonstrated significant value not only as a reactive source of silica and alkalis but also as a high-temperature stabilizer in blended cementitious systems. Dai et al. [26] reported that glass powder enhanced thermal resistance, refined microstructure, and preserved compressive strength in oil well cement pastes cured at elevated temperatures offering insights that may be adapted for geopolymer systems requiring high thermal durability.

Table 5 presents a comprehensive comparative analysis of the chemical compositions of various geopolymer precursor materials, encompassing industrial by-products, agricultural residues, and naturally occurring minerals. These materials were evaluated for their oxide content, which directly influences their pozzolanic activity, alkali activation potential, and gel-forming behavior in geopolymer systems.

1. Silica Content (SiO_2_) and Amorphous Potential

Silicon dioxide (SiO_2_) is the principal component responsible for forming geopolymeric networks, particularly sodium aluminosilicate hydrate (N–A–S–H) gels. Materials such as silica fume (96.9%), rice husk ash (83.10%), rice straw ash (69.20%), and waste glass (69.65%) possess exceptionally high amorphous silica content, enabling rapid dissolution and polymerization. Agricultural ashes, including bamboo leaf ash (72.97%) and sugarcane bagasse ash (76.00%), also exhibit >70% SiO_2_ when processed under controlled burning conditions, ensuring high pozzolanic reactivity. Coal gasification fly ash (CGFA, 61.30%), though slightly lower in silica, offers significant amorphous content suitable for alkali-activated systems, provided its heavy metal variability is carefully managed.

2. Alumina Content (Al_2_O_3_)

Aluminum oxide contributes to the tetrahedral framework that complements silica in geopolymer binders, influencing gel structure and mechanical strength. Fly ash (25.8%), desulfurization waste from titanium slag (DWTS, 48.21%), kaolinite (32.60%), and zeolite (11.71%) show moderate-to-high alumina contents, demonstrating strong geopolymerization potential. CGFA (24.94% Al_2_O_3_) is also emerging as an alternative aluminosilicate precursor, with comparable reactivity to conventional fly ash. DWTS, due to its exceptionally high Al_2_O_3_, is increasingly considered a viable alternative to metakaolin, though its long-term durability and optimal activation conditions require further study.

3. Calcium Oxide (CaO) and C–A–S–H Gel Potential

High-CaO precursors such as ground granulated blast furnace slag (GGBS, 40.45%) and granulated phosphorus slag (GPS, 47.47%) promote the formation of calcium-aluminosilicate-hydrate (C–A–S–H) and calcium-silicate-hydrate (C–S–H) gels, enhancing early-age strength and reducing permeability. CGFA, with 1.11% CaO, functions better in low-calcium hybrid systems where the primary binding phase remains aluminosilicate-based (N–A–S–H). Moderate CaO levels in bamboo leaf ash (6.07%) and basalt powder (7.50%) make them suitable for hybrid AAS–ASP formulations where both N–A–S–H and C–A–S–H gels coexist.

4. Iron Oxide (Fe_2_O_3_) and Chromophoric Effects

Precursors such as red mud (8.71%), tektite (8.40%), fly ash (8.40%), and basalt powder (15.00%) contain significant Fe_2_O_3_, which can influence color, thermal mass, and microstructure. Although Fe_2_O_3_ is not the primary network former, it contributes to (Fe)–A–S–H gel formation, increasing density, thermal stability, and chemical resistance. Iron-rich laterites and basalt powders, in particular, have demonstrated compatibility with aluminosilicate phosphate (ASP) systems, making them promising for high-temperature and fire-resistant applications.

5. Alkali Content (Na_2_O + K_2_O)

High intrinsic alkali content facilitates aluminosilicate dissolution, reducing dependence on commercial activators. Waste glass (Na_2_O: 9.69%, K_2_O: 0.39%) and bamboo leaf ash (Na_2_O: 1.23%, K_2_O: 6.07%) can act as both reactive precursors and self-activating agents. CGFA, with low intrinsic alkalis (Na_2_O: 0.09%, K_2_O: 1.48%), requires external alkaline activators for effective geopolymerization. Alkali-poor materials such as kaolinite and bentonite similarly demand supplementary alkaline solutions to achieve high reactivity.

6. Sulfur and Titanium Oxides

Elevated TiO_2_ in DWTS (0.51%), GPS (0.22%), basalt powder (2.00%), and red mud (2.70%) enhances UV resistance and, in some cases, photocatalytic properties. SO_3_, present in GGBS (4.74%), DWTS (1.70%), and gold mine tailings (3.05%), requires careful mix design to mitigate risks of sulfate attack or expansive reactions, particularly in wet or marine environments.

7. Emerging and Alternative Precursors

The increasing inclusion of bamboo leaf ash, basalt powder, rice straw ash, waste glass, and CGFA reflects a global shift toward low-cost, regionally available, and renewable precursors. CGFA is particularly relevant as a substitute for fly ash, given its high aluminosilicate content, though leaching of trace heavy metals must be controlled through mix design and encapsulation strategies. Such alternative precursors are crucial due to the projected decline in fly ash and slag availability as coal-fired power plants are phased out. Moreover, iron-rich laterites, basalt powders, and phosphorus slag have demonstrated excellent compatibility with ASP systems, further broadening their application potential, including in cold asphalt pavement systems where GGBS and calcium carbide residue have been shown to enhance mechanical performance [120]. Recent performance evaluations confirm that these alternative precursors can reduce carbon emissions while maintaining mechanical efficiency [21]. However, standardized processing and comprehensive durability studies remain limited, representing key future research gaps.

### 2.9. Chemical Pathways in Geopolymerization: The Role of Hardeners, Alkalination, and Activation

The study of geopolymer technology is increasingly vital to the advancement of sustainable construction practices. Central to this field is a clear understanding of hardeners, along with the important distinction between alkali activation and alkalination. These chemical processes significantly affect the performance and durability of geopolymers and are fundamental to the development of eco-friendly material alternatives [57,68]. Geopolymerization relies heavily on hardeners, which are crucial for the chemical transformation of aluminosilicate materials. Typically, these hardeners are alkaline, playing a pivotal role in the synthesis of geopolymers. They contribute to the depolymerization of aluminosilicate precursors and raise the pH, creating optimal conditions for polymerization [68]. This depolymerization breaks covalent bonds, releasing reactive monomers such as aluminates and silicates, which then organize into the geopolymeric network [29].

The success of this chemical reaction is influenced by the type and concentration of the alkaline substance employed. Alkaline hardeners encompass a variety of compounds, including hydroxides, silicates, carbonates, and sulfates from alkali metals like sodium, potassium, and lithium. Among these, hydroxides such as NaOH, KOH, and LiOH are notably popular due to their high reactivity [121,122]. Silicates, including sodium silicate and potassium silicate, also frequently apply in the process. For effective geopolymerization, the pH of the solution needs to generally surpass 11.5. It is important to note that while increasing concentrations of alkalis (in the range of 4–14 M) can enhance mechanical strength [123], exceeding this range might lead to diminished performance due to increased viscosity or gel formation.

Considering the environmental and economic challenges posed by commercially sourced alkaline activators, there is a growing interest in exploring alternative hardeners derived from local or waste materials. Alternative sources such as pumice [124] and agricultural waste like rice husk ash [104] offer eco-friendly options for activator synthesis. Biomass ashes such as those from rice husks, almond shells, and hazelnut shells waste glass and quartz have all shown potential for producing alkali silicates through fusion processes [125,126]. Although these methods align well with the principles of waste valorization and a circular economy, the environmental costs associated with high-temperature fusion processes raise important questions [85,87].

A key area of discussion within this field is the differentiation between “alkali activation” and “alkalination.” Though often treated as synonymous, these terms refer to distinct processes [46,57]. Alkali activation broadly describes a chemical method aimed at enhancing the reactivity of a material typically by improving surface area, porosity, or the number of reactive sites which is applicable in various areas, including the production of activated carbon and catalysts. Alkalination, on the other hand, specifically addresses the introduction of alkaline substances to stimulate chemical reactions, primarily by modifying pH or promoting transformation [46]. In the context of geopolymer science, alkalination provides a more precise description of how aluminosilicate precursors react with alkaline solutions to yield solid binders [47,127].

Recognizing this distinction is crucial; misinterpretation of these terms can obscure the underlying chemistry and obstruct efforts to enhance geopolymer formulations. By clarifying that geopolymerization more accurately reflects the process of alkalination, researchers can engage in more rigorous scientific discourse and innovation. Furthermore, there is potential in acidic geopolymerization, although it is less commonly explored. Phosphoric acid, for instance, has emerged as a well-studied acidic hardener [128]. It interacts with aluminosilicate precursors like metakaolin to form a network through the bonding of tetrahedral phosphate (PO_4_) and aluminum-oxygen (Al–O) units. These acidic geopolymers demonstrate high compressive strength, thermal stability, and favorable dielectric properties [29], making them versatile materials with applications that extend beyond construction to areas such as catalytic reduction of nitrogen oxides (NOx) [75,92,128]. By accurately detailing the chemical mechanisms involved in geopolymer synthesis, especially focusing on the roles of hardeners and the nature of alkalination, researchers can devise more efficient and sustainable material solutions. This clarity not only corrects longstanding misconceptions in existing literature but also paves the way for enhancing geopolymer technology in alignment with principles of green chemistry and sustainable development [48,129].

### 2.10. Optimizing Precursor Reactivity in Geopolymer Synthesis

The reactivity of aluminosilicate materials is a crucial factor governing the successful synthesis and performance of geopolymer binders. Several interrelated parameters influence this reactivity, with chemical composition particularly the silicon-to-aluminum (Si/Al) ratio playing a pivotal role. Generally, a higher Si/Al ratio is associated with an increased dissolution rate and improved polycondensation kinetics, resulting in geopolymers that exhibit enhanced mechanical properties and long-term durability [78].

Alongside composition, physical characteristics such as particle size, structural morphology, and pre-treatment techniques (e.g., thermal or mechanical activation) significantly affect reactivity. Smaller particle sizes offer a greater surface area for reactions, thereby enhancing surface reactivity and promoting better interaction with activating solutions. Techniques such as grinding, ultrafine milling, and calcination are commonly employed to increase surface area and induce structural transformation. For example, calcination can convert crystalline phases in clay into amorphous, more reactive phases, particularly transforming kaolinite into metakaolin, thereby facilitating the breakdown of rigid aluminosilicate frameworks [57].

Chemical treatments may also eliminate impurities that inhibit dissolution or interfere with the geopolymerization process, further enhancing reactivity [130]. Recent advances show that combining ultrafine grinding with thermal activation significantly boosts pozzolanic reactivity. Wei et al. [131] found that thermally activated ultrafine recycled fine powder (TAURFP) produced denser matrices and superior mechanical properties compared to untreated or singly treated powders.

Kaolinitic clays with more than 50% kaolinite content, when thermally treated at around 700 °C, convert almost entirely into metakaolin a notably reactive phase while preserving inert quartz [132]. The early-stage reactivity of metakaolin is typically evaluated by using performance indices such as compressive strength benchmarking against standard mortars, affirming its suitability as a precursor [130,132]. As primary aluminosilicate sources, clay minerals like kaolinite possess inherent reactivity due to their layered architecture and recurring aluminosilicate bonding configurations, such as [≡Si–O–Al–O]_n_ and [≡Si–O–Al–O=]_n_. These structures are loosely held via electrostatic forces, yielding a high specific surface area for chemical interaction. Their ion exchange capacity and hydration-induced swelling/shrinkage behavior enhance their versatility across engineering and environmental applications [57,133].

In contrast, secondary aluminosilicate materials like fly ash and ground granulated blast furnace slag (GGBFS) primarily consist of amorphous, glassy phases. These materials are known for their high pozzolanic activity and favorable Si/Al ratios, making them highly reactive in alkaline environments. Upon activation with hydroxide-based solutions such as sodium hydroxide (NaOH) or potassium hydroxide (KOH), these materials dissolve and form geopolymer gels—critical binding phases in the hardened matrix [130].

Geopolymerization of aluminosilicate precursors can follow two principal chemical pathways. The alkaline route uses hydroxides and soluble alkali silicates to form gel networks composed of N–A–S–H or C–A–S–H structures. Alternatively, the acidic route involves phosphoric acid activation, resulting in poly(aluminophospho) gel structures with distinct thermal and dielectric properties [92].

In addition to empirical methods, thermodynamic modeling has emerged as a valuable tool for optimizing precursor reactivity and predicting product formation. Modeling frameworks such as GEMS, FactSage, and Thermo-Calc simulate the phase assemblages formed under varying precursor compositions, activator concentrations, and curing conditions. This approach is particularly effective in CaO–SiO_2_–Al_2_O_3_ systems, where multiple overlapping phase transitions occur. Such simulations can predict the formation of amorphous versus crystalline gels, stability ranges of key phases, and the influence of factors like Si/Al and Ca/Si ratios on reaction kinetics and product durability [40]. Integrating thermodynamic modeling with experimental mix design not only reduces the trial-and-error process but also enables more targeted use of both natural and industrial precursors.

Ultimately, understanding and optimizing the reactivity of both primary (natural) and secondary (industrial) aluminosilicates is essential for tailoring geopolymer binder systems to specific performance and sustainability goals. Strategies involving precursor refinement, compositional tuning, and predictive modeling provide a scientific basis for mix design, helping accelerate the adoption of geopolymers in construction and other advanced material applications. These approaches align with broader objectives of reducing environmental impact and promoting material circularity [57,78].

## 3. Engineering Geopolymer Properties Through Molar Ratio Design

### 3.1. Optimizing Molar Ratios

In geopolymer technology, the balance of key molar ratios governs network polymerization, gel-phase formation, and long-term durability. The SiO_2_/Al_2_O_3_ ratio already introduced in Section 1.4 (basic system distinctions: N–A–S–H vs. C–A–S–H gels) is critical for tailoring structural and chemical properties. Phase-level implications of these ratios are further elaborated in Section 2.4. Silicon and aluminum primarily derived from fly ash, slag, and metakaolin serve as essential network formers [79,134,135]. Adjusting this ratio using tailored precursor blends and supplementary silica additives significantly modifies dissolution kinetics, setting, and strength development [136,137].

The SiO_2_/Al_2_O_3_ ratio governs key performance parameters:

- Polymer Network Formation: Higher silica content promotes dense Si–O–Al and Si–O–Si linkages, improving compressive strength and microstructural integrity [135,136]. Xu et al. [136] reported enhanced sulfate resistance due to reduced pore connectivity.
- Viscosity and Workability: As detailed in Section 2.4, higher Si/Al ratios increase viscosity, improving moldability but reducing early workability; optimized ratios in fly ash–slag blends balance rheology and strength [57,89,138].
- Setting and Strength Development: Lower ratios accelerate setting and early strength, whereas higher ratios enhance long-term mechanical stability. Recent results indicate an optimal range of 3.4–3.8 for achieving balanced early and ultimate strength [57,137,139,140]. Zhang et al. [137] reported that alkali-activated geopolymer cement mortar optimized within this range showed superior trench backfilling performance due to its compact microstructure.
- Durability: Densely cross-linked networks with higher silica content improve thermal stability, chemical resistance, and freeze–thaw durability [57,136,141].

Other molar ratios also play significant roles in the geopolymerization process (see Section 2.4 for detailed gel interactions):

- M_2_O/SiO_2_ and M_2_O/Al_2_O_3_: Critical for regulating the alkaline environment and precursor dissolution, ensuring stable polymerization [142,143].
- H_2_O/M_2_O: Governs dissolution kinetics and gel formation, directly affecting workability and final strength [144,145].
- SiO_2_/Na_2_O: Governs network connectivity and mechanical resilience, with higher ratios promoting microstructural homogeneity and reduced pore connectivity [101,142].

Iron-Rich Systems:

- SiO_2_/Fe_2_O_3_: Moderate Fe incorporation enhances nucleation and refines the gel matrix, whereas excess Fe disrupts network cohesion [83].
- Na/Fe and Al/Fe Ratios: Regulate porosity, phase stability, and thermal resistance; higher Al/Fe ratios are particularly advantageous in acidic or sulfate-rich environments [83].
- Strategic Molar Design: By interlinking these ratios, tailored binders can be engineered for chemically aggressive, thermally extreme, or structurally demanding applications. Furthermore, a nonlinear three-component creep model originally proposed for polymer-alloy geocell sheets [146] has been suggested as a transferable predictive framework for modeling time-dependent deformation in geopolymer composites.

### 3.2. Influence of Activator Chemistry

Alkaline activator chemistry governs precursor dissolution, gel nucleation, and polymer network densification. The general reaction framework of activators was outlined in Section 1.4, whereas Section 2.4 explains their role in gel phase chemistry (N–A–S–H and C–A–S–H evolution). Conventional activators alkaline hydroxides (NaOH, KOH) and silicates (sodium or potassium silicate) are strongly governed by molarity and the silicate modulus (SiO_2_/M_2_O) [47,48,136]. Optimizing NaOH concentrations (8–12 M) ensures sufficient dissolution without inducing rapid gelation, shrinkage, or microcracking, which are common at excessively high molarities [30,137,142].

The SiO_2_/M_2_O ratio significantly affects polycondensation and gel densification. Increased silicate content enhances long-term strength and durability through refined cross-linking; however, very high ratios delay setting and reduce early strength [47,136,139]. Tailored silicate moduli have been shown to improve pore structure refinement and sulfate resistance in fly ash–slag systems [136].

The choice of alkali cation influences gel structure and thermal behavior: Na^+^-based activators form denser N–A–S–H or C–A–S–H gels at lower cost, while K^+^-based activators provide better thermal stability due to their larger ionic radius, making them ideal for fire-resistant applications [13,48,147]. Mixed systems (NaOH + sodium silicate) balance early strength and workability [137,140].

Sustainable Activator Systems: Alternative activators derived from rice husk ash and biomass ashes can deliver comparable mechanical and durability performance while significantly reducing CO_2_ emissions. RHA-synthesized sodium silicate has demonstrated refined pore structures and long-term durability equivalent to conventional activators [104,125].

### 3.3. Effect of Curing Regimes

Curing regimes directly influence reaction kinetics and gel phase evolution, as explained mechanistically in Section 2.4. Temperature, humidity, and duration dictate precursor dissolution rates, gel densification, and pore refinement [30,114]. Elevated-temperature curing accelerates precursor dissolution and gel formation, achieving higher early-age strength. Optimal conditions for fly ash– and metakaolin-based geopolymers are typically 60–80 °C for 24–48 h [30,114,148]. However, excessive temperatures can induce shrinkage and microcracking, necessitating controlled thermal profiles [15]. Recent findings show that carefully optimized heat curing enhances pore refinement and reduces permeability, directly improving sulfate resistance and freeze–thaw durability [136].

Ambient curing is increasingly favored for sustainable and large-scale applications despite slower strength development. Studies indicate that adjusting activator concentration and silicate modulus can compensate for slower reaction kinetics, allowing ambient-cured mixes to achieve competitive long-term strengths [56,137].

Relative humidity also governs reaction kinetics: low humidity promotes drying shrinkage and microcracking, whereas excessive moisture can dilute the activator, hindering geopolymerization [15]. Sealed or moisture-retentive curing has been recommended to balance these effects and ensure durable matrix formation [15,148].

Innovative curing methods including steam curing, microwave-assisted curing, and carbonation curing have shown potential to accelerate gel phase evolution, refine pore structures, and improve resistance to chemical attack [149,150]. These methods are particularly valuable for rapid infrastructure applications where early demolding and service loading are required.

### 3.4. Reaction Kinetics and Gel Phase Evolution

Reaction kinetics and gel phase evolution, detailed in Section 2.4, are key to understanding the transformation from aluminosilicate dissolution to stable gel networks. In the initial stage, aluminosilicate precursors dissolve in an alkaline medium, liberating reactive silicate and aluminate species. Subsequent polycondensation leads to the formation of amorphous N–A–S–H (sodium aluminosilicate hydrate) gels in low-calcium systems and mixed N–A–S–H/C–A–S–H (calcium aluminosilicate hydrate) gels in high-calcium systems [39,46,84]. Xu et al. (2024) [136] demonstrated that controlling dissolution kinetics through optimized activator molarity and precursor fineness significantly refines the N–A–S–H network, reducing pore connectivity and improving sulfate resistance.

Influencing Factors: The reaction rate is primarily governed by activator concentration, precursor fineness, and curing temperature. Elevated alkalinity and finer particles accelerate dissolution, resulting in rapid gel nucleation and early-age strength development. However, excessively rapid kinetics can lead to shrinkage-induced microcracking, especially in thermally cured systems [83,127,150].

Gel Phase Evolution: Over time, residual unreacted precursors continue to dissolve, enabling secondary gel formation and further densification of the matrix [47,123]. In slag-rich systems, additional C–A–S–H phases intermix with N–A–S–H, enhancing compressive strength and reducing permeability [39]. However, excessive Ca incorporation can destabilize the aluminosilicate network, compromising chemical durability [47,137].

Emerging studies on Fe-rich geopolymers show that Fe can substitute for Al within the gel framework, modifying gel morphology and improving thermal resistance, though high Fe content may retard reaction kinetics [83]. Zhang et al. [137] further highlighted that fine-tuning calcium and iron ratios is essential for balancing early strength with long-term chemical stability, particularly in aggressive sulfate or chloride environments.

### 3.5. Microstructural Development and Porosity Control

Microstructural refinement, closely linked to gel phase behavior discussed in Section 2.4, determines porosity, strength, and long-term durability. The amorphous N–A–S–H and C–A–S–H gel phases interconnect to form a dense three-dimensional framework, encapsulating partially reacted particles and reducing pore continuity [30,84,123]. Negahban et al. [151] demonstrated that a refined gel network significantly decreases mesopore connectivity, improving resistance to chloride ingress and freeze–thaw cycles.

Porosity and Mechanical Performance: Lower total porosity correlates directly with improved compressive strength and chemical durability. Fine control of precursor fineness and activator concentration accelerates dissolution, promoting homogeneous gel distribution and minimizing capillary voids [127,136]. However, excessively rapid reactions may trap unreacted particles, generating weak interfacial transition zones (ITZs) and microcracking [83].

Calcium and Pore Structure: High-calcium blends exhibit reduced capillary porosity due to secondary C–A–S–H gel infilling, which complements the N–A–S–H matrix [39]. Yet, over-incorporation of calcium can destabilize the gel network, inducing localized shrinkage and heterogeneity [47,137].

Curing and Pore Refinement: Properly optimized curing balancing temperature and humidity facilitates gradual densification. Xu et al. (2024) [136] confirmed that staged heat curing (moderate early heat, followed by ambient curing) significantly decreases large pore fractions while maintaining microstructural stability. Conversely, abrupt high-temperature curing can induce thermal microcracking [150].

Advanced Strategies: Incorporating reactive nano-silica or supplementary aluminosilicates further refines the pore structure, while Zhang et al. (2025) [137] emphasized that tailored calcium–silica ratios are key for achieving durable microstructures in sulfate-rich environments.

Long-term durability is pivotal for ensuring the reliable performance of geopolymers in structural and environmental applications. The aluminosilicate framework, when properly optimized, exhibits superior resistance to chloride and sulfate attack, freeze–thaw cycling, and thermal fluctuations compared to Portland cement systems [15,56]. Durability is strongly governed by the Si/Al, Na/Al, and H_2_O/Na_2_O ratios, which regulate gel cross-linking and pore connectivity, thereby reducing leaching and chemical degradation over extended service periods [57,136,141]. Microstructural densification through optimized curing regimes further improves impermeability and chemical stability [137].

Predictive approaches, including life cycle assessment (LCA) and AI-based modeling, are increasingly used to evaluate long-term performance. Ramesh et al. [19] applied AI-integrated LCA to estimate both durability trends and carbon footprint reductions, while Ji et al. [73] demonstrated that optimized molar ratios significantly reduce emissions and enhance performance in road infrastructure. Incorporating such predictive tools alongside pore structure evolution studies [136] provides a robust basis for forecasting service life, particularly for applications in sulfate-rich soils, marine environments, and thermally aggressive conditions.

### 3.6. Long-Term Durability and Performance Prediction

Predicting the long-term performance of geopolymers requires integrating microstructural parameters (as detailed in Section 3.5) with advanced durability modeling techniques. While dense N–A–S–H and C–A–S–H gel networks enhance resistance to chloride ingress, sulfate attack, and thermal fluctuations, quantifying these effects over decades demands robust predictive frameworks.

1. Microstructure-Driven Service-Life Models

The refined pore structures achieved through optimized curing and tailored Ca–Si ratios (Section 3.5) directly influence permeability and ion transport, which are primary indicators of service life. Xu et al. [136] emphasized that reduced mesopore connectivity correlates with lower diffusivity, allowing service-life models to incorporate measurable parameters such as pore size distribution, tortuosity, and moisture retention.

2. Life Cycle Assessment (LCA) Integration

AI-enhanced LCA approaches are emerging as powerful tools to estimate both environmental and durability performance. Ramesh et al. [19] demonstrated that integrating LCA with durability data enables simultaneous prediction of carbon footprint reduction and structural longevity, essential for sustainable infrastructure planning.

3. Performance-Based Predictive Tools

Ji et al. [73] highlighted that optimized molar ratios (as established in Section 3.1) reduce maintenance cycles in road infrastructure, providing a quantifiable basis for service-life extension. Similarly, machine learning models trained on microstructural and chemical datasets are being developed to forecast degradation rates under aggressive sulfate, chloride, or freeze–thaw exposure.

4. Reliability in Harsh Environments

By combining pore refinement data (Section 3.5) with environmental exposure models, predictive frameworks can simulate geopolymer performance in sulfate-rich soils, marine environments, and thermally aggressive conditions. These models increasingly utilize time-dependent creep and shrinkage simulations, with Zhao et al. [146] nonlinear creep model originally developed for polymer-alloy composites being explored as a transferable method for geopolymer composites.

## 4. Structural Chemistry and Bonding in Geopolymer Networks

Geopolymers are three-dimensional aluminosilicate frameworks that bridge amorphous and semi-crystalline phases, primarily structured by Q^4^ units consisting of SiO_4_ tetrahedra. These tetrahedra are linked via Si–O–Si and Si–O–Al bonds, forming a continuous network whose structural complexity depends on the Si/Al ratio (Section 3.1) and gel chemistry (Section 2.4). Charge imbalances introduced by Al^3+^ substitution for Si^4+^ are neutralized by alkali or alkaline-earth cations such as Na^+^, K^+^, Ca^2+^, and Fe^3+^, which influence network stability and durability (Section 3.5) [57,147,152].

**Figure 8:**
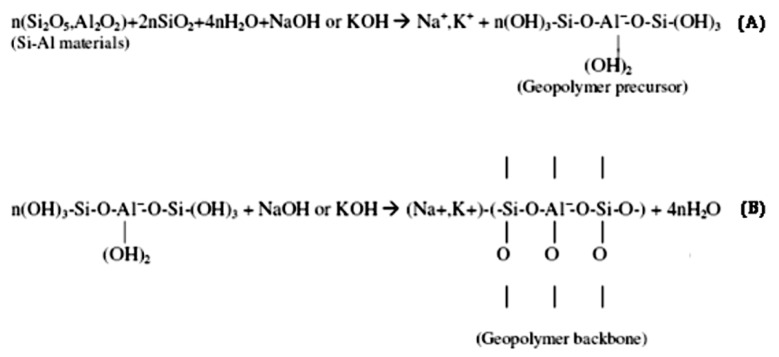
Chemical Processes Underlying Geopolymer Synthesis [153].

The reaction mechanism (Figure 8) begins with alkaline dissolution of aluminosilicate precursors, generating silicate and aluminate species that progressively condense into a cross-linked aluminosilicate framework. This process detailed mechanistically in Section 1.4 and Section 2.4 is governed by precursor reactivity and activator chemistry (Section 3.2).

A. Hydroxide ions (OH^−^) attack Si–O–Si and Al–O–Si bonds, releasing reactive monomeric species. The dissolution rate depends strongly on precursor fineness and activator molarity, as previously established (Section 3.4).
B. Dissolved species align and condense into monomers, which polymerize into stable sialate and sialate-siloxo networks. Their connectivity ranging from simple poly(sialate) to highly cross-linked poly(sialate-disiloxo) directly governs microstructural densification and long-term durability (Section 3.5).

Geopolymerization proceeds via dissolution, condensation, and polycondensation of reactive silicate and aluminate species, ultimately forming a rigid aluminosilicate network. The substitution of Al^3+^ for Si^4+^ introduces negative charges, stabilized by alkali or alkaline-earth cations (Na^+^, K^+^, Ca^2+^), which also affect pore structure and chemical resistance (Section 3.5) [152].

**Figure 9:**
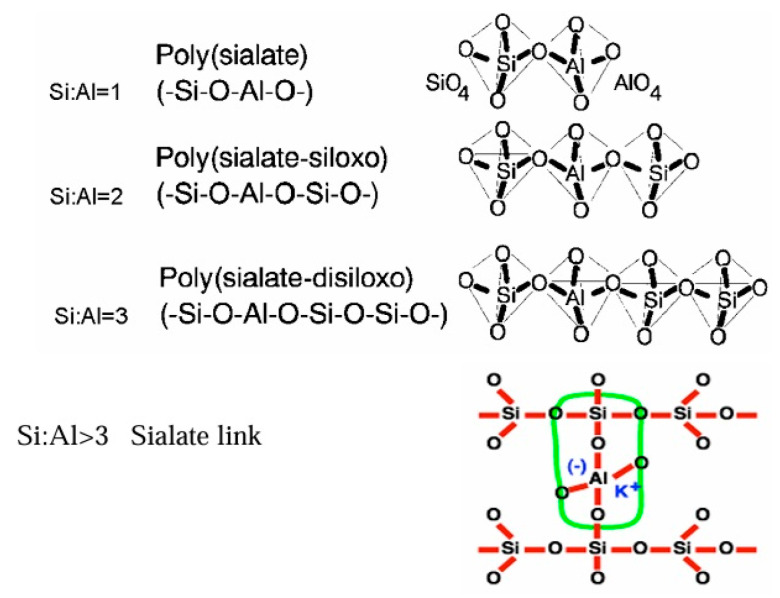
The Geopolymerization Process: The Polysialate formation [155].

Framework complexity is dictated by Si/Al ratios (Section 3.1) and gel-phase transitions (Section 2.4), which produce diverse molecular configurations ranging from low-silica poly(sialate) to high-silica poly(sialate-disiloxo) networks [154]. These units have been included in Table 6 as follows:

The structural chemistry of geopolymers is intrinsically linked to their silicon-to-aluminum (Si:Al) ratio, which dictates the type of polymeric units formed, their degree of cross-linking, and consequently their mechanical and durability characteristics. Figure 9 presents the fundamental structural classifications of geopolymer networks based on these Si:Al ratios, highlighting the chemical composition and connectivity of different units such as poly(sialate), poly(sialate-siloxo), and poly(sialate-disiloxo). These frameworks are distinguished by the number of silicate units bridging aluminosilicate tetrahedra, which directly influences the degree of polymerization and final material properties. The figure also illustrates a sialate link with charge-balancing cations (e.g., Na^+^ or K^+^), which stabilize the three-dimensional aluminosilicate network.

**Figure 10:**
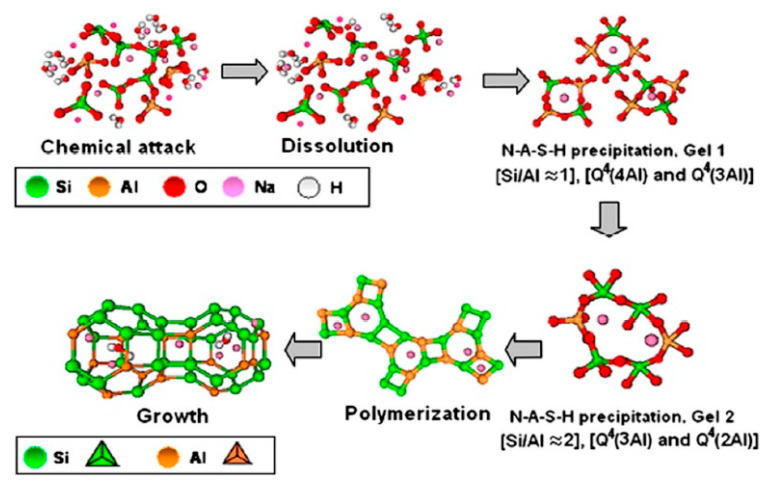
The Geopolymerization Process [30] [© 2006 Springer. Adapted with permission].

Building on these structural units, Figure 10 depicts the molecular-scale transformation processes during geopolymer gel formation. Beginning with the chemical attack and dissolution of aluminosilicate precursors, the process advances through N–A–S–H gel precipitation, polymerization, and growth of the three-dimensional aluminosilicate network. This stepwise transformation explains how the structural units shown in Figure 10 evolve during geopolymerization to form a highly cross-linked, durable network.

**Figure 11:**
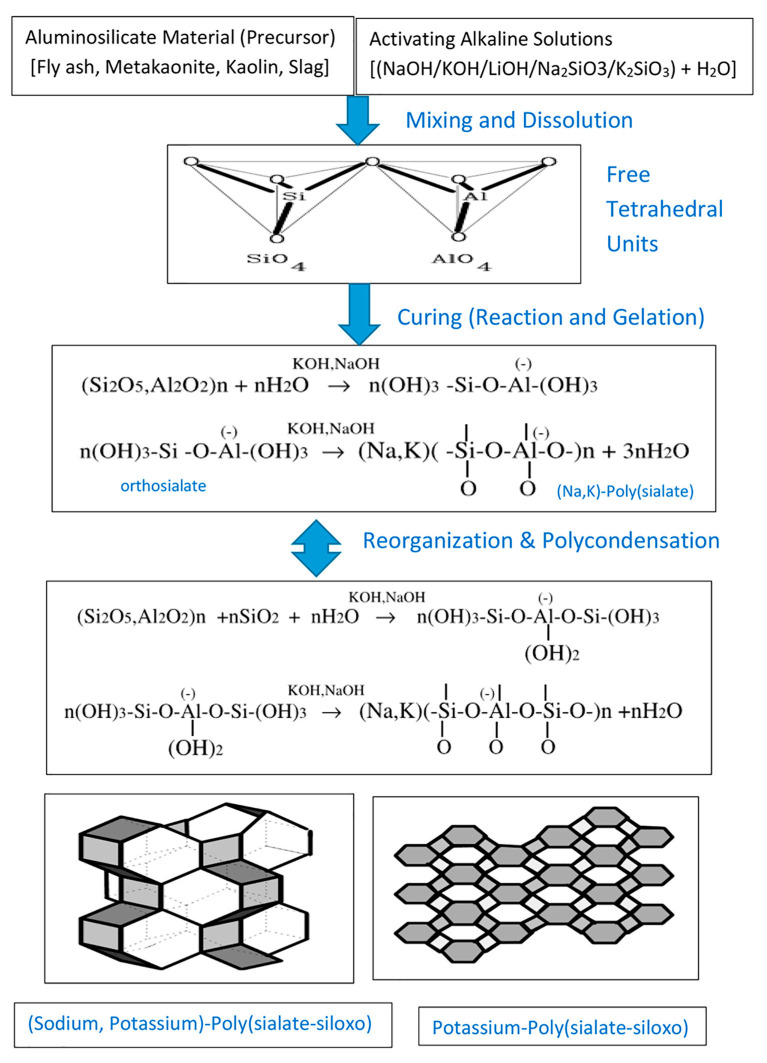
Geopolymerization Process of Alkali Aluminosilicate (AAS) Geopolymers: Mixing, Dissolution, and Polycondensation Pathways [10].

Figure 11 illustrates the complete geopolymerization pathway, beginning with the dissolution of aluminosilicate precursors (such as fly ash, metakaolin, or slag) in highly alkaline solutions. This process breaks down the original structure, liberating reactive silica and alumina species which then undergo gelation to form initial polysialate units. Through subsequent reorganization and polycondensation reactions, a three-dimensional aluminosilicate framework is formed often described as N–A–S–H gel resulting in a hardened geopolymer matrix with distinct molecular architecture such as (Na,K)-poly(sialate-siloxo) networks. The figure also highlights key intermediate species and chemical transitions occurring throughout this multistage transformation.

The polycondensation reactions that govern geopolymer formation fundamentally distinguish them from Ordinary Portland Cement (OPC). Unlike OPC, which relies on the hydration of crystalline clinker phases (C_3_S, C_2_S, C_3_A, and C_4_AF) to form calcium silicate hydrate (C–S–H) and calcium hydroxide (CH) [149], geopolymers are formed through covalent bonding between SiO_4_ and AlO_4_ tetrahedra in a largely amorphous aluminosilicate matrix [152]. This cross-linked three-dimensional network, stabilized by charge-balancing cations, results in low porosity, chemical inertness, and resistance to aggressive agents such as sulfates common deterioration factors in OPC-based systems. Moreover, recent interface characterization tools, such as pull-off and fluorescence tracing methods [156], provide deeper insight into geopolymer–aggregate bonding, which is crucial for optimizing durability in moisture-sensitive environments. These structural and bonding mechanisms not only enhance the long-term performance of geopolymers but also position them as promising alternatives for sustainable infrastructure applications.

### 4.1. Classification and Chemistry of Acid-Based Geopolymers

Geopolymers are increasingly recognized for their unique properties and potential applications in construction and materials science. They can be categorized primarily based on the reaction medium utilized during synthesis. The two principal classes are alkali-aluminosilicate (AAS) geopolymers and aluminosilicate phosphate (ASP) geopolymers. AAS geopolymers are produced through the reaction of aluminosilicate materials with alkaline activators, while ASP geopolymers emerge from synthesis in acidic environments, typically involving phosphoric acid or phosphate-based solutions [76]. Both classes harness aluminosilicate sources like metakaolin, volcanic ash, and various industrial by-products, such as blast furnace slag [57].

However, the pathways of their chemical reactions and the resultant network structures distinguish them significantly. In ASP systems, the incorporation of phosphorus atoms modifies the silicate and aluminate framework, leading to the formation of a unique silicoaluminophosphate network [52]. The synthesis of ASP geopolymers typically involves a dual-component approach, which includes an aluminosilicate precursor and an acid phosphate solution, giving rise to what are often called acid-based or phosphate geopolymers. The formation process is activated by the extraction of cations from the precursor, followed by the release of anions, which together fosters the development of a three-dimensional binder network [147].

The primary structural units within these systems consist of tetrahedral [PO_4_]^5−^, [AlO_4_]^3−^, and [SiO_4_]^4−^ groups. Interestingly, ASP geopolymerization can take place under ambient conditions. For instance, mixtures such as monoaluminum phosphate (MAP) combined with metakaolin at a near 1:1 Al/P molar ratio yield a predominantly amorphous binder with moderate strength. This structure may also contain crystalline phases, such as AlH_3_(PO_4_)_2_·3H_2_O. However, it is important to note that this amorphous structure is thermally unstable above 210 °C, progressively transitioning to more stable crystalline forms like quartz and berlinite (AlPO_4_), which enhances compressive strength [74,76].

The structural properties of ASP geopolymers are contingent upon the composition of the precursor and the processing parameters. For example, systems employing fly ash, glass powder, and phosphoric acid might yield crystalline phases like brushite and monetite alongside amorphous silicophosphate and silicoaluminophosphate gels [147]. Conversely, when metakaolin is the precursor, the resulting matrix frequently comprises berlinite and amorphous aluminosilicophosphate components [74,76].

Typically, temperatures below 100 °C produce amorphous binders, while synthesis at around 50 °C under high humidity can facilitate the crystallization of aluminum phosphate phases [76]. Optimal performance in ASP systems that use metakaolin is achievable at specific formulations and curing conditions. Research suggests that a P/Al ratio of approximately 0.6, with curing at 50 °C and 98% relative humidity for seven days, promotes high mechanical strength due to a favorable charge distribution between phosphate and aluminate species [147].

There is also potential for ASP systems to utilize alternative aluminosilicate materials, such as lateritic and iron-rich clays. When calcined at 600 °C and react with phosphoric acid (pH ≤ 2), these materials demonstrate significant compressive strength development as they mature over time [57,128]. For instance, after 28 days of curing at 40 °C, iron-rich laterite (LAI) achieved a compressive strength of 65 ± 1 MPa, whereas standard laterite (LAC) reached 52 ± 1 MPa. The enhanced iron content in LAI plays a critical role in its improved mechanical properties in acidic contexts [57]. ASP geopolymers created in acidic environments tend to form dense matrices with low porosity, characterized by complex crystalline and semi-crystalline phases, including berlinite (FePO_4_), iron hydrogen phosphate hydrate, and ferrowyllieite. These phases testify to the rich geochemical interactions throughout the polymerization process, particularly in systems that utilize alternative raw materials [74,76].

The formation of ASP geopolymers generally unfolds through three discernible stages:

(1) Dealumination, instigated by phosphoric acid, which breaks Al–O–Al and Si–O–Al bonds to liberate reactive species [75,157];
(2) Polycondensation among PO_4_^3−^, Al^3+^, and silicate components, resulting in the generation of alumino- and silico-phosphate gel networks, with AlPO_4_ crystals also forming as secondary phases [158];
(3) Network establishment, characterized by continued condensation and crystallization, resulting in a hybrid matrix comprising both amorphous and crystalline domains [74,157].

Despite the intricacies associated with their reaction pathways and the more stringent synthesis conditions required, ASP geopolymers offer distinctive advantages over their alkali-activated counterparts. They exhibit superior thermal stability, enhanced mechanical strength, and improved dielectric properties [80,81]. These beneficial attributes position ASP geopolymers as promising candidates for advanced construction applications and high-performance composite materials [147].

### 4.2. The Chemistry of Alkali Aluminosilicate (AAS) Geopolymers

Alkali aluminosilicate (AAS) geopolymers represent a significant advancement in materials science, achieved through intricate molecular transformations that yield durable, three-dimensional polymeric frameworks. These frameworks are crucial for the structural and functional performance of the final material [76]. A comprehensive understanding of these chemical processes is essential to optimize geopolymer synthesis and tailor their properties for innovative construction and environmental applications [37,76].

The geopolymerization process begins with the dissolution of silicon (Si) and aluminum (Al) species from aluminosilicate precursors, such as metakaolin and fly ash, under highly alkaline conditions. Hydroxide ions break Si–O–Si and Al–O–Si bonds, releasing reactive silicate and aluminate monomers into the solution [159]. These species then undergo orientation and condensation reactions, progressively forming oligomers and eventually evolving into complex polymeric networks. A critical early step involves alkalination and depolymerization of the aluminosilicate source, which generates tetravalent aluminum species within the sialate group (–Si–O–Al(OH)_3_^−^Na^+^).

This multistage transformation, beginning with the dissolution of aluminosilicate precursors and progressing through gelation and polycondensation, is schematically illustrated in Figure 11. The figure highlights the sequential chemical reactions and intermediate gel phases that ultimately lead to the formation of a hardened three-dimensional aluminosilicate network.

Pentavalent sialate structures subsequently undergo cleavage, transferring electrons from silicon to oxygen, and forming reactive intermediates such as silanol (Si–OH) and basic siloxo (Si–O^−^) groups [160,161]. These intermediates then evolve into ortho-sialate units, which are key structural components in the geopolymerization mechanism [160]. Concurrently, basic siloxo groups coordinate with sodium cations (Na^+^), forming stable Si–O–Na bonds, thereby strengthening the developing aluminosilicate network.

**Figure 12:**
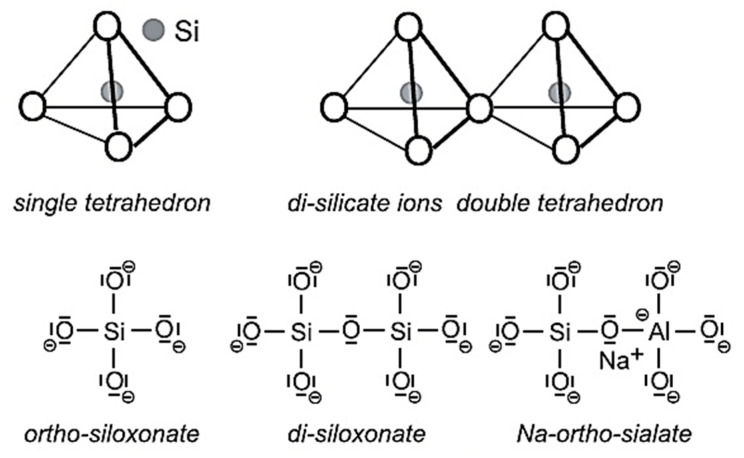
Representative Structural Units in AAS Geopolymers: Tetrahedral Configurations and Key Siloxo/Sialate Species [6].

As the reaction progresses, ortho-sialate species (Si–ONa) condense with aluminum hydroxyl groups (OH–Al), producing cyclo-tri-sialate rings and releasing NaOH into the system. The liberated alkali further accelerates polycondensation, facilitating the formation of a Na-poly(sialate) nepheline-type framework [159]. The introduction of water glass (sodium silicate solution) enhances these processes by stimulating additional reactions with ortho-sialate species, di-siloxane Q1 species, and reactive groups (Si–ONa, Si–OH, and OH–Al). The molecular-level arrangement of these structural units, particularly the tetrahedral coordination of silicon and aluminum and their covalent bonding via sialate and siloxo linkages, is illustrated in Figure 12. This figure demonstrates how simple tetrahedral units evolve into di-silicate and sialate structures, which eventually polymerize into a dense cross-linked aluminosilicate network. Such tetrahedral connectivity is fundamental to the superior mechanical strength, chemical resistance, and thermal durability of AAS geopolymers. This synergy promotes the development of ortho-sialate-disiloxo cyclic structures while generating more NaOH, which further encourages cross-linking [159,160].

The final phase of geopolymerization is characterized by extensive polycondensation, culminating in a highly organized Na-poly(sialate-disiloxo) framework. This crystalline-like network features a feldspar-like crankshaft chain structure, endowing AAS geopolymers with high mechanical stability, superior chemical resistance, and excellent thermal durability [159]. The distinctive bonding mechanisms underscore the critical role of alkali chemistry in creating high-performance, sustainable geopolymeric materials for diverse engineering and environmental applications.

The fundamental difference between acid-based (ASP) and alkali aluminosilicate (AAS) geopolymers lies in their reaction pathways, structural networks, and performance attributes. ASP systems rely on dealumination and phosphate-driven polycondensation, producing a silicoaluminophosphate network that often contains crystalline phases such as berlinite (AlPO_4_) and brushite, giving them superior thermal stability and dielectric properties [54,76,80]. In contrast, AAS geopolymers form through alkaline dissolution, sialate-polycondensation, and disiloxo cross-linking, yielding N–A–S–H or (Na,K)-poly(sialate-siloxo) networks with feldspar-like crankshaft chain structures [6,159,160]. While ASP systems excel in high-temperature and chemically aggressive environments, AAS geopolymers demonstrate faster setting, higher early mechanical strength, and easier scalability in construction applications [37,76,147]. Thus, understanding the distinct molecular mechanisms of these systems is crucial for tailoring geopolymers to specific engineering and environmental applications.

## 5. Geopolymers for Construction and Environmental Applications: A Comparative Study of the Alkali Aluminosilicate (AAS) and Aluminosilicate Phosphate (ASP)

The following presents a detailed comparison of alkali aluminosilicate (AAS) and aluminosilicate phosphate (ASP) geopolymers, emphasizing their unique advantages, challenges, and potential applications across various domains, including environmental, biomedical, and technological fields. The following discussion synthesizes key parameters associated with these two types of geopolymers. Environmental Impact and Production AAS geopolymers generally have a higher environmental impact, largely due to the energy-intensive process required for synthesizing sodium silicate, a key activator [104,109,124]. This process can lead to significant greenhouse gas emissions and utilize non-recyclable materials, raising concerns about environmental sustainability [20].

In contrast, ASP geopolymers offer a more environmentally friendly profile. They are produced through low-temperature reactions involving phosphate rock and sulfuric acid, resulting in modest CO_2_ emissions. Furthermore, these reactions can facilitate the recovery and reuse of leftover phosphorus, aligning principles of ecological sustainability and circular economy [76]. Cost and Availability AAS geopolymers benefit from affordable feedstocks, primarily fly ash and blast furnace slag, making them easily accessible for large-scale construction and infrastructure projects [25,162].

However, ASP geopolymers may entail higher production costs due to requiring specialized reagents, such as phosphoric acid. Additionally, their accessibility may vary by geographic region, influencing their scalability and implementation [76,81]. Construction Applications AAS geopolymers are well-suited for structural and infrastructure applications due to their excellent compressive strength, durability, and cost efficiency. They are commonly utilized in precast components, pavements, and structural composites [36,163].

Conversely, ASP geopolymers excel in specialized applications requiring thermal insulation and fire resistance, making them ideal for refractory linings and fire-retardant coatings. Their low dielectric constant also permits usage in high-temperature electrical environments [76,81]. Biomedical Applications The application of AAS geopolymers in biomedical settings is somewhat limited, primarily due to their basic chemical nature and the potential for leaching harmful ions from constituents like fly ash and slag, which are not biocompatible [52,63]. On the other hand, ASP geopolymers demonstrate promising biocompatibility and tunable reactivity, making them suitable candidates for biomedical applications, including bioactive scaffolds, bone repair materials, and drug delivery systems [52,63,98]. 3D Printing Potential AAS geopolymers have shown effective results in large-scale additive manufacturing processes for construction elements, with workability and setting times adjustable through activator concentration and temperature variations [99,100].

ASP geopolymers, meanwhile, exhibit significant potential for precision applications, such as 3D bioprinting and the fabrication of medical devices, owing to their room-temperature reactivity and adjustable setting behavior [52,63,98,99]. Waste Encapsulation and Management Both AAS and ASP geopolymers excel in immobilizing toxic elements, including heavy metals and radio nuclides. AAS geopolymers are frequently employed in environmental remediation efforts [59]. In contrast, ASP geopolymers are similarly proficient at encapsulating hazardous and radioactive waste, aided by their structural stability in thermal and acidic environments, ensuring long-term containment [97].

Curing Conditions and Processing Sensitivity For optimal strength development, AAS geopolymers require moderately elevated curing temperatures (ranging from 40 to 90 °C) and are sensitive to curing duration and moisture levels [148,150]. Conversely, ASP geopolymers can effectively cure at ambient or slightly elevated temperatures. Their reactivity is highly influenced by phosphate concentration and environmental humidity, thus allowing for energy-efficient processing [164,165]. Durability in Aggressive Environments AAS geopolymers are known for their resilience in alkaline, saline, and marine environments, although they may suffer degradation under prolonged acidic exposure [15]. In contrast, ASP geopolymers demonstrate superior performance in acidic and high-temperature conditions, offering enhanced resistance to environmental degradation [76].

Microstructure and Porosity, AAS geopolymers typically develop moderately porous matrices, with their microstructure significantly affected by the type and concentration of the alkali activator used [136]. On the other hand, ASP geopolymers create compact, low-porosity networks with structural integrity strengthened through homogeneous polymerization controlled by humidity and phosphate levels during curing [151].

Sustainability and Circular Economy, While AAS geopolymers support the valorization of industrial by-products, they face challenges in fully achieving sustainability due to their reliance on synthetic sodium silicate [20]. Conversely, ASP geopolymers align well with circular economy practices through phosphorus recovery, low-carbon production processes, and potential agricultural reuse of waste products [166].

Standardization and Research Maturity, AAS geopolymers have established a robust framework in research and industry contexts, supported by numerous case studies, field trials, and increasing adoption under emerging ASTM and ISO standards [167]. In contrast, ASP geopolymers are gaining traction in scientific literature but require further development in mix design standards, performance benchmarks, and commercial-scale pilot testing to enhance their applicability [76]. This comparative analysis sheds light on the distinctive properties of AAS and ASP geopolymers, providing a valuable resource for researchers and practitioners seeking to enhance the use of geopolymers in various applications while addressing sustainability challenges.

## 6. Performance and Application-Based Comparison of AAS and ASP Geopolymers

The relationship between alkali aluminosilicate (AAS) and aluminosilicate phosphate (ASP) geopolymers reveal significant advantages and challenges, shaped by their environmental impact, cost considerations, and geopolymers demonstrate functional adaptability across a wide range of industries from conventional construction to advanced biomedical engineering. Among them, alkali-activated slag (AAS) geopolymers are particularly favored for typical construction uses due to their performance and material availability, primarily due to the widespread availability and cost-effectiveness of precursor materials such as fly ash and slag. Their synthesis processes are well-established, making them practical for large-scale infrastructure projects [56,168]. On the other hand, ASP geopolymers stand out for their enhanced environmental sustainability and thermal stability. Such properties position them as ideal candidates for specialized uses, particularly in contexts where ecological performance or high-temperature resistance is critical, such as in fireproofing solutions or medical devices [76].

### Phase Chemistry and the Structural Role of Gel Phases (N–A–S–H, K-A-S-H, C-S-H, and C–A–S–H) in Geopolymers

The core difference between geopolymer cement (GPC) and ordinary Portland cement (OPC) lies in their chemical reaction pathways and the binding phases they produce. OPC develops strength mainly through hydration, resulting in the formation of calcium silicate hydrate (CSH) gel. The introduction of aluminum-rich additives, like slag or fly ash, can initiate the formation of calcium alumino-silicate hydrate (C–A–S–H), further enhancing durability and mechanical strength [96]. In contrast, geopolymer concrete (GPC) is synthesized via geopolymerization, which forms a three-dimensional aluminosilicate framework [8,160]. While C–S–H-like phases may develop in calcium-rich formulations especially those incorporating slag they are not the primary binding agents within the geopolymer matrix. Instead, intermediate gels such as sodium alumino-silicate hydrate (NASH) and potassium alumino-silicate hydrate (KASH) form during the early stages of the geopolymerization process [147]. These phases later progress into a more stable, densely cross-linked aluminosilicate framework. It is important to note that alkali-activated materials (AAMs), which can sometimes be misclassified as geopolymers, often remain stuck in the NASH or KASH gel phases. This limitation can lead to issues of solubility and leaching, adversely affecting their long-term performance [39]. To develop genuine geopolymer characteristics, the system should be augmented with network-enhancing components, such as metakaolin (MK-750) [86], which promote the creation of a durable and chemically stable structure [169]. This distinction aligns with the prevailing scientific consensus that geopolymers are essentially low-calcium alkali-activated materials dominated by N–A–S–H or K-A-S-H gels, whereas high-calcium alkali-activated systems characterized by C–A–S–H and C-S-H gels fall outside the strict definition of geopolymers and are instead categorized as alkali-activated binders [46,48]. Metakaolin plays a crucial role in establishing a robust three-dimensional framework by surrounding alkali cations (Na^+^, K^+^), thereby reducing leachability and bolstering mechanical strength [137,170]. The inclusion of calcium and iron-based gels aids in densifying the microstructure by filling voids and decreasing porosity, leading to improved interparticle contact and mechanical performance [171]. In conclusion, while gel phases like CSH are central to the OPC system, they do not constitute the primary structural components in geopolymers. Misinterpreting these gel phases as definitive products in GPC could result in inaccurate evaluations of material performance. It is the hardened aluminosilicate network, the stable framework that provides strength and durability to geopolymer systems. Recognizing this fundamental difference is vital for the accurate assessment and optimization of geopolymers in diverse engineering applications. Recent thermodynamic and phase assemblage modeling studies in CaO–SiO_2_–Al_2_O_3_ systems have demonstrated that careful precursor selection and reaction product management can significantly influence gel formation and stability, reinforcing the need for predictive design tools in geopolymer chemistry [40].

## 7. Conclusions

This review has critically examined the fundamental and emerging aspects of geopolymer chemistry, with particular attention to molecular mechanisms, gel and phase evolution, and the influence of design parameters on performance. By differentiating alkali-aluminosilicate (AAS) and aluminosilicate phosphate (ASP) systems, the review establishes a chemically rigorous framework for understanding activation processes, gel structures, and material functionality. Key compositional ratios, particularly Si/Al, Na_2_O/SiO_2_, and H_2_O/M_2_O, have been shown to govern mechanical behavior, setting kinetics, and long-term durability.

Unlike previous reviews, which largely emphasize conventional AAS systems based on fly ash or slag, this work provides an integrated comparison of both AAS and ASP systems, explicitly linking phase chemistry (N–A–S–H, K-A-S-H, C-S-H, and C–A–S–H gels) with structural roles and performance attributes. This dual-system approach is complemented by discussions on underexplored precursors such as iron-rich laterites, basalt powders, waste glass, coal gasification fly ash, and phosphoric-acid-activated clays, offering a broader material perspective that extends beyond the usual bibliographic content.

Furthermore, the valorization of industrial by-products and incorporation of circular economy principles are discussed in the context of emerging trends, including AI-assisted mix optimization, hybrid waste valorization, and multifunctional composite systems such as CICF-reinforced geopolymers. These perspectives highlight the growing interdisciplinarity of the field and demonstrate how geopolymer materials can transition from experimental research to sustainable infrastructure solutions.

Although this review spans a wide thematic range, this approach is deliberate to ensure that identified research gaps such as multifunctional geopolymer matrices and hybrid waste valorization are situated within a comprehensive and comparative framework of geopolymer science and application. In doing so, the review provides a differentiated and forward-looking synthesis that addresses not only the established AAS domain but also emerging ASP systems and novel waste-derived formulations, thus filling a key gap in existing literature.

## 8. Future Considerations

Based on this synthesis, several underexplored but promising research directions emerge that merit further investigation. Notably, the integration of thermodynamic modeling and phase assemblage simulations has been highlighted as a critical emerging tool for managing reaction products and optimizing precursor design in CaO–SiO_2_–Al_2_O_3_ systems [40], offering a predictive framework that can guide future mix designs for both AAS and ASP systems. Four critical areas identified are:

1. Carbon Fiber and Advanced Fiber Reinforcements—The incorporation of fiber reinforcements, particularly chromium-impregnated carbon fibers (CICFs) derived from tannery buffing dust, presents significant potential to enhance the tensile strength, flexural toughness, and crack resistance of alkali aluminosilicate (AAS) and aluminosilicate phosphate (ASP) geopolymers. Although not addressed in the current literature covered in this review, CICF represents a promising future direction due to its dual functionality: improving mechanical properties while immobilizing chromium and other toxic metals. This aligns with the broader sustainability and waste valorization strategies highlighted in Section 1, Section 2 and Section 5. Moreover, hybrid waste stream integration, involving the co-utilization of by-products and hazardous or organic waste sources from nature, industry, or agriculture, could further expand resource availability and improve binder performance.
2. Underexplored Industrial and Secondary Raw Materials—Future studies should systematically investigate a broader range of precursors beyond the conventional fly ash and slag. Promising materials include waste glass (high amorphous silica content, enhancing N–A–S–H gel formation), coal gasification fly ash (CGFA) (rich in reactive aluminosilicates, though heavy metal variability requires careful assessment), iron-rich laterites and basalt powders (notable for their dense crystalline phases and excellent performance in ASP systems), and red mud (bauxite residue), which offers potential for hybrid alkali–acid activation. Additionally, phosphorus slag and rice husk ash (RHA) present sustainable options for incorporating calcium, phosphorus, and silica-rich phases, while waste ceramics and demolition waste powders provide low-cost, readily available aluminosilicate sources for large-scale applications. Integrating these diverse secondary materials aligns with circular economy principles and can significantly expand the performance envelope of both AAS and ASP systems.
3. Hybrid Waste Stream Integration—Expanding on the principles of circular economy highlighted in Section 1 and Section 5, future work should explore hybrid systems that co-utilize diverse waste streams including agricultural residues, hazardous industrial by-products, and organic wastes. Controlled interaction between such waste streams and aluminosilicate matrices could yield multifunctional binders with improved chemical stability, reduced porosity, and enhanced performance under aggressive environments.
4. Development of Multifunctional Geopolymer Matrices—Emerging applications increasingly demand engineered binder systems capable of fulfilling structural, chemical, and environmental functions. Future research should target ASP systems for biomedical and high-temperature applications and AAS systems for structural and environmental remediation contexts. Designing these matrices to perform waste immobilization, heavy metal stabilization, and chemical durability enhancement will require fine-tuned control of gel phases (N–A–S–H, K-A-S-H, and C–A–S–H) and network cross-linking mechanisms (Section 6). The application of thermodynamic modeling and phase assemblage predictions, as demonstrated by [40], could be instrumental in anticipating stable gel phase formation and optimizing precursor combinations for multifunctional matrices.

A promising integrated pathway involves optimizing such systems through molar ratio tuning, fiber–matrix interface design, and long-term durability assessment under realistic environmental exposures. The synergy of these approaches may establish geopolymers, especially CICF-reinforced and hybrid waste-based composites as next-generation, multifunctional materials for low-carbon construction, environmental remediation, and advanced industrial applications.

## Figures and Tables

**Table 1:** Environmental Impact Assessment of Raw Materials in One-Part Geopolymers [20].

Geopolymer						OPC
Ingredients kg (per kg of binder)	**Fly Ash**	**GGBS**	**Sodium Silicate**	**Sodium Carbonate**	**Total**	
	0.5	0.32	0.09	0.09	1	
Carbon emission (kg CO_2_-e/kg)	0.0135	0.0457	0.0803	0.0225	0.162	0.86
Embodied energy (MJ/kg)	0.05	0.1056	1.611	0.1215	1.888	5.6

**Table 2:** Key Differences between Geopolymers and High-Calcium Alkali-Activated Materials (AAMs) [11,39,46,47,48].

Binder Type	Main Reaction Gel	Ca Content	Typical Precursors	Primary Durability Focus
Geopolymers—Low-Ca AAM	N–A–S–H (sodium aluminosilicate hydrate)	<5–10 wt% Ca	Fly ash, metakaolin, waste glass, red mud ^1^	ASR risk, drying shrinkage
High-Ca AAM (e.g., Slag-Based)	C–A–S–H (calcium aluminosilicate hydrate)	>10 wt% Ca	GGBS, slag-rich blends, limestone-rich by-products	Carbonation, sulfate and acid attack

^1^ Red mud composition varies by source. While primarily used as a low-calcium aluminosilicate precursor, some red muds contain moderate CaO levels that may contribute to minor C–A–S–H gel formation, placing them at the boundary between low- and medium-Ca alkali-activated materials [49,50,51].

**Table 3:** Geopolymers and Alignment with Key United Nations Sustainable Development Goals (SDGs).

SDG	Geopolymer Contribution	Key Research Gaps	Citation
SDG 9—Industry, Innovation, and Infrastructure	Development of resilient precast infrastructure, pavements, and advanced Alkali-Activated Slag—Alkali-Activated Silicophosphate systems for structural applications.	Long-term structural performance under varied climatic and loading conditions.	[15,52,53]
SDG 12—Responsible Consumption and Production	Valorization of industrial by-products (fly ash, GGBS, rice husk ash, waste glass, red mud, coal gasification fly ash).	Precursor variability, consistent quality control, and supply chain sustainability.	[9,11,12,25]
SDG 13—Climate Action	Up to 80% CO_2_ reduction compared to OPC; Life Cycle Assessment studies confirm significant environmental benefits.	Comprehensive life-cycle assessment for emerging precursors (waste glass, CGFA, red mud).	[16,17,18,19,60]

**Table 4:** Comparative SEM and XRD analysis of major industrial and emerging precursors.

Precursor	SEM Observations (Morphology)	XRD Observations (Phase Characteristics)	Microstructural Implications	Citation
Fly Ash (FA)	Predominantly spherical, smooth glassy microspheres; some porous cenospheres	Dominant amorphous hump (20–35° 2θ); crystalline quartz, mullite, hematite peaks	Spherical morphology enhances workability; high amorphous phase improves dissolution and geopolymerization	[108]
GGBS	Irregular, angular, rough particles; glassy texture visible	Broad amorphous hump (25–35° 2θ); minor crystalline merwinite and akermanite	High Ca amorphous phase accelerates C–A–S–H gel formation; irregular morphology slightly lowers flowability	[103,104,109]
Metakaolin (MK)	Plate-like, flaky particles with high surface area	Semi-crystalline; quartz and muscovite peaks; amorphous aluminosilicate derived from dehydroxylated kaolinite	High surface area and amorphous content enhance reactivity; improves strength and durability	[108]
Red Mud (RM)	Agglomerated, irregular plate-like particles; dense clusters	Crystalline hematite, goethite, perovskite, gibbsite; low amorphous content	High Fe_2_O_3_ & CaO contribute to (Fe)-A-S-H or C–A–S–H gel formation; excessive Fe reduces workability	[49,51]
Waste Glass (WG)	Angular, sharp-edged particles; smooth fractured surfaces	Broad amorphous hump (20–30° 2θ); minor crystalline quartz and wollastonite	High reactive silica improves polymerization; angular morphology may need alumina-rich blends for balance	[11,26]
Coal Gasification Fly Ash (CGFA)	Finer than FA; mixed cenospheres and angular grains	High amorphous content; crystalline quartz, mullite, magnetite	High reactivity and fine size accelerate gel formation; durable performance still under research	[12]
Bamboo Leaf Ash (BLA)	Irregular porous particles; fibrous ash texture	High amorphous silica (~73%); minor crystalline quartz	High silica improves N–A–S–H gel formation; porous morphology increases water demand	[90]
Rice Husk Ash (RHA)	Fine, highly porous particles; honeycomb-like structure	Broad amorphous silica hump; crystalline cristobalite if over-burnt	High reactive silica boosts polymerization; porous structure may influence workability	[93,104]
Rice Straw Ash (RSA)	Flaky, irregular porous ash particles	High amorphous SiO_2_ (~69%); some quartz peaks	Similar to RHA; promising as low-cost precursor; moderate CaO enables hybrid C–A–S–H gel potential	[91]
Basalt Powder (BP)	Angular, dense, rough particles	Moderate amorphous content; crystalline plagioclase and pyroxene	Moderate CaO (7–8%) supports hybrid binder systems; dense morphology may reduce flowability	[112]

**Table 5:** Chemical Composition of Precursors.

Precursors	Chemical Composition (%)									Citation
	SiO_2_	Al_2_O_3_	Fe_2_O_3_	CaO	MgO	Na_2_O	K_2_O	TiO_2_	SO_3_	
Fly ash	52.4	25.8	8.4	6.42	2.27	-	1.47	1.31	0.86	[95]
Ground Granulated Blast Furnace Slag	32.19	13.89	0.35	40.45	6.67	0.28	0.32	0.74	4.74	[95,113]
Desulfurization Waste from Titanium Slag	33.07	48.21	6.74	2.94	1.48	0.28	1.21	0.51	1.70	[113]
Kaolinite	51.30	32.60	1.10	0.10	0.30	0.20	0.30	1.10	0.00	[114]
Rice Husk Ash	83.10	2.15	1.10	4.70	1.50	0.10	2.96	-	1.20	[93]
Pumice	75.23	14.04	1.95	0.52	0.22	2.09	5.05	0.11	0.29	[115]
Silica Fume	96.9	0.15	0.06	0.53	1.1	-	0.78	-	0.12	[95]
Sugarcane Bagasse Ash	76.00	9.00	4.20	3.10	2.70	-	3.83	0.46	-	[14]
Tektite	69.84	12.16	8.40	2.54	2.03	1.07	2.28	0.78	-	[33]
Alccofine	37.53	24.57	0.92	29.46	5.23	0.03	0.61	-	0.18	[116]
Red Mud	29.18	30.01	8.71	15.96	0.89	8.22	0.80	2.70	2.73	[51]
Waste Glass	69.65	0.85	0.50	14.45	2.89	9.69	0.39	0.10	0.55	[11]
Coal gasification fly ash	61.30	24.94	3.86	1.11	0.67	0.09	1.48	2.01	0.021	[12]
Gold Mine Tailings	74.50	6.98	7.03	0.53	5.16	0.27	1.26	0.44	3.05	[117]
Zeolite	70.92	11.71	0.92	2.10	0.33	2.06	3.09	-	-	[118]
Bentonite	68.10	15.44	0.34	0.77	3.79	2.58	0.93	0.12	-	[118]
Basalt Powder	45.00	15.00	15.00	7.50	5.20	3.00	1.00	2.00	-	[112]
Bamboo Leaf Ash	72.97	2.85	2.31	4.98	1.23	-	6.07	0.41	0.55	[90]
Rice straw ash	69.20	5.30	0.90	3.46	2.81	3.43	6.40	-	-	[91]
Granulated Phosphorus Slag	39.11	2.38	0.74	47.47	4.60	0.03	0.15	-	0.22	[119]

**Table 6:** Framework and their Polymeric Description.

Framework Type	General Formula	Polymeric Description	Citations
Siloxo	–Si–O–Si–O–	Poly(siloxo)	[6,70]
Sialate	–Si–O–Al–O–	Poly(sialate)	[6,70]
Sialate-siloxo	–Si–O–Al–O–Si–O–	Poly(sialate–siloxo)	[6,70]
Sialate-disiloxo	–Si–O–Al–O–Si–O–Si–O–	Poly(sialate–disiloxo)	[6,70]
Phosphate	–P–O–P–O–	Poly(phosphate)	[6]
Phospho-siloxo	–P–O–Si–O–P–O–	Poly(phosphor–siloxo)	[6]
Phospho-sialate	–P–O–Si–O–Al–O–P–O–	Poly(phosphor–sialate)	[6]
Alumino-phospho	–Al–O–P–O–	Poly(alumino–phospho)	[6]
Ferro-sialate	–Fe–O–Si–O–Al–O–Si–O–	Poly(ferro–sialate)	[6]

## Data Availability

No new data were created or analyzed in this study. Data sharing is not applicable to this article.

## Abbreviations

The following abbreviations are used in this manuscript:

2θ	Diffraction Angle
AAMs	Alkali-Activated Materials
AAS	Alkali Aluminosilicate
AI	Artificial Intelligence
ANNs	Artificial Neural Networks
ANFIS	Adaptive Neuro-Fuzzy Inference System
ASP	Aluminosilicate Phosphate
ASR	Alkali–Silica Reaction
Al	Aluminum
BLA	Bamboo Leaf Ash
BP	Basalt Powder
CGFA	Coal Gasification Fly Ash
C–A–S–H	Calcium Aluminosilicate Hydrate
CH	Calcium Hydroxide
CICF	Chromium-Impregnated Carbon Fibers
CSH	Calcium Silicate Hydrate
CaO	Calcium Oxide
DNN	Deep Neural Network
DWTS	Desulfurization Waste from Titanium Slag
FA	Fly Ash
FeO	Ferrous Oxide
GEP	Gene Expression Programming
GGBFS	Ground Granulated Blast Furnace Slag
GPC	Geopolymer Concrete
GPS	Granulated Phosphorous Slag
IGCC	Integrated Gasification Combined Cycle
KASH	Potassium Aluminosilicate Hydrate
KOH	Potassium Hydroxide
LAC	Lateritic Aluminosilicate Clay
LAI	Lateritic Aluminosilicate (Iron-rich)
LCA	Life Cycle Assessment
MAP	Monoaluminum Phosphate
MK-750	Metakaolin Calcined at 750 °C
ML	Machine Learning
NASH	Sodium Aluminosilicate Hydrate
NOx	Nitrogen Oxides
NaOH	Sodium Hydroxide
OH	Hydroxide Ion
OPC	Ordinary Portland Cement
ResNet	Residual Network
RF	Random Forest
RHA	Rice Husk Ash
RM	Red Mud
RSA	Rice Straw Ash
SBA	Sugarcane Bagasse Ash
SCMs	Supplementary Cementitious Materials
SDGs	Sustainable Development Goals
SEM	Scanning Electron Microscopy
Si	Silicon
Si/Al	Silicon-to-Aluminum Ratio
SVM	Support Vector Machine
WG	Waste Glass
XRD	X-ray Diffraction
